# Unilateral Versus Bilateral Percutaneous Kyphoplasty for Single-Level Thoracolumbar Osteoporotic Vertebral Compression Fractures: A Systematic Review and Meta-Analysis

**DOI:** 10.3390/jcm15187030

**Published:** 2026-09-10

**Authors:** Panagiotis Korovessis, Vasileios Syrimpeis, Georgios Vlachopoulos, Dimitrios Ntourantonis, George Sakellaropoulos

**Affiliations:** 1 Olympion General Hospital of Patras, 26443 Patras, Greece; vsyrimpeis@gmail.com; 2 Department of Medical Physics, School of Medicine, University of Patras, 26110 Patras, Greece; gvlachop@upatras.gr (G.V.); gsak@upatras.gr (G.S.); 3 Sigma Delta Analytics, 26110 Patras, Greece; 4 Emergency Department, University Hospital of Patras, 26443 Patras, Greece; d_douradonis@yahoo.gr

**Keywords:** osteoporotic vertebral compression fracture, percutaneous kyphoplasty, vertebral augmentation, balloon kyphoplasty, unilateral approach, bilateral approach, meta-analysis, systematic review

## Abstract

**Background/Objectives:** The optimal surgical approach for Percutaneous KyphoPlasty (PKP) in patients with **recent single-level Osteoporotic Vertebral Compression Fractures** (OVCFs) remains controversial. Most available previous meta-analyses included studies with variable heterogeneity, often mixing unilateral and bilateral MIS approaches, differing surgical techniques, and various fracture patterns, which limited the reliability of their conclusions. This meta-analysis aimed to compare the efficacy and safety of unilateral versus bilateral PKP exclusively in patients with recent single-level OVCFs only. **Methods:** A systematic review was conducted according to the PRISMA 2020 guidelines. PubMed, Scopus, Cochrane Library, and ScienceDirect were searched for comparative studies published between 2000 and 2025. Randomized Controlled Trials (RCTs), prospective, and retrospective comparative studies comparing unilateral and bilateral PKP for recent single-level OVCFs were included. Clinical and radiological outcomes as well as perioperative complications and safety outcomes were analyzed using random-effects meta-analysis. Predefined subgroup analyses according to study design and sensitivity analyses were performed. **Results:** Eleven studies involving 1374 patients (705 unilateral and 669 bilateral PKP) met the inclusion criteria. No significant differences were observed between the two surgical approaches regarding short- or long-term pain relief, cement leakage, number of adjacent vertebral fractures, or overall clinical outcomes. Bilateral PKP demonstrated statistically significant, but clinically negligible, advantages in anterior vertebral body height restoration and kyphosis correction. Unilateral PKP required an insignificantly lower cement volume. For operative time, the overall pooled estimate favored unilateral PKP by approximately 10 min but showed extreme heterogeneity (I^2^ = 98.5%). Importantly, the two RCTs showed no statistically significant between-group difference (MD = +1.2 min, 95% CI −4.5 to +6.8), indicating that the apparent overall effect was largely driven by observational evidence. Similar discrepancies between randomized and retrospective studies were observed for several other outcomes, underscoring the importance of considering study design when interpreting the results. **Conclusions:** Current evidence does not demonstrate clinically meaningful superiority of either unilateral or bilateral PKP for the treatment of recent single-level OVCFs. Bilateral PKP may provide small advantages in selected radiographic outcomes, whereas unilateral PKP uses modestly less bone cement; however, the relevance of these differences remains clinically uncertain. Surgical approach selection may therefore be individualized according to vertebral morphology, pedicle anatomy, fracture characteristics, surgeon experience, and technical feasibility rather than expectations of superior clinical outcomes. Further adequately powered randomized trials with standardized outcome reporting and long-term follow-up are warranted.

## 1. Introduction

Osteoporotic Vertebral Compression Fractures (OVCFs) in the thoracolumbar spine are a hallmark complication of postmenopausal osteoporosis, serving as a primary driver of debilitating back pain, progressive kyphotic deformity, and mortality among elderly populations [1,2,3,4]. Beyond structural spinal instability, the systemic sequelae of OVCFs—including restricted mobility, impaired pulmonary function, and profound psychological distress—frequently lead to a loss of independent living in elderly patients [5]. While traditional conservative management involves bed rest, analgesia, and external bracing, these strategies often result in prolonged, often unpredictable recoveries [6,7]. Consequently, Percutaneous VertebroPlasty (PVP) and Percutaneous balloon KyphoPlasty (PKP), have become the clinical standard to stabilize the fractured osteoporotic vertebral body, alleviate associated pain, and restore vertebral body height after failed conservative therapy [8]. Nevertheless, clinical consensus regarding the absolute short- and long-term efficacy and safety profiles of these procedures remains elusive due to persistent heterogeneity across the published literature [9,10,11,12,13,14,15,16].

Debate persists regarding whether PKP should be executed via a unilateral or bilateral transpedicular approach. The standard bilateral approach is favored for facilitating symmetric polymethylmethacrylate (PMMA) distribution, whereas a unilateral approach raises theoretical concerns regarding asymmetric cement distribution and subsequent suboptimal mechanical restoration and permanent stability [17]. Conversely, accumulated evidence indicates that both transpedicular augmentation approaches yield comparable clinical scores (e.g., Visual Analog Scale [VAS], Oswestry Disability Index [ODI]) and overall complication rates, without the higher risks of soft tissue injury, neural damage, or cement leakage historically feared with bilateral approaches [18]. Owing to advantages like shorter operative times, reduced intraoperative blood loss, and minimized radiation exposure, the unilateral technique has gained popularity [7]. These benefits are particularly pronounced in single-level OVCFs where the index vertebra features consistent biomechanics, whereas multilevel pathologies demonstrate distinct radiographic features, accelerated bone fragility, and altered spinal mechanical stability that confound treatment responses [19,20,21].

Over the past 12 years, sixteen meta-analyses have attempted to synthesize the comparative outcomes of transpedicular unilateral versus bilateral PKP and PVP approaches [7,22,23,24,25,26,27,28,29,30,31,32,33,34,35,36]. However, the pooled analyses in these meta-analyses exhibit substantial confounding heterogeneity because they often mix PKP with PVP cases, and single-level with and multilevel interventions [21,22,37,38]. Crucial radiographic parameters such as vertebral body height restoration, correction of the kyphotic Cobb angle, cement leakage, and adjacent-level fractures are most reliable when they are evaluated at an isolated, single treated segment. Including multilevel procedures introduces major confounding variables by naturally escalating total cement volume, lengthening operative exposure, and compounding leakage risks [22,23].

Additional sources of heterogeneity across previous studies may include differences in puncture technique and trajectory, fracture characteristics and vertebral morphology, and patient-related factors such as age, bone mineral density, baseline clinical status, and fracture level. These factors may influence treatment selection, cement distribution, radiographic correction, and clinical outcomes, thereby complicating direct comparisons between unilateral and bilateral approaches.

To address these limitations, we conducted a systematic review and meta-analysis specifically restricted to direct comparisons of unilateral and bilateral PKP in recent, single-level OVCFs. Restriction to recent OVCFs reduces heterogeneity related to fracture chronicity, healing stage, and structural remodeling, while restriction to a single treated vertebral level minimizes the procedural variability associated with multilevel interventions, including differences in cement requirements, operative exposure, and technical complexity. This more clinically homogeneous population allows a more focused comparison of the two PKP approaches and improves the applicability of the findings to the clinical decision of selecting unilateral or bilateral access for an individual recent osteoporotic vertebral fracture. Unlike previous meta-analyses that frequently combined different vertebral augmentation techniques and/or single-level with multilevel fractures, the present study also evaluates randomized and observational evidence separately according to study design, aiming to provide a more precise assessment of whether either PKP technique offers a clinically meaningful advantage.

## 2. Materials and Methods

### 2.1. Literature Search Strategy

Following PRISMA (Preferred Reporting Items for Systematic Reviews and Meta-Analyses) guidelines [39], a systematic search was conducted across PubMed, Scopus, Cochrane and ScienceDirect to identify studies published between 2000 and 2025. The search was designed to identify all potentially eligible studies using the following query:

(“*kyphoplasty*” OR “*vertebropl*asty” OR “*spinal augmentation*” *OR* “*osteoporotic fracture*” OR “*unipedicular*” OR “*bipedicular*”).

This search strategy retrieved all records containing at least one of the aforementioned search terms. In total, 9.563 records were identified and subsequently screened for eligibility.

Only studies published in English or available in an English translation were included.

The criteria for including studies in this review were based on the **PICO** [40] (Population, Intervention, Comparison, and Outcome) framework.

**Population (P)**: Adult patients (>40 years) with single-level thoracic or lumbar osteoporotic vertebral compression fractures (OVCFs).

**Interventional treatment (I)**: Unilateral Percutaneous Kyphoplasty.

**Comparison-Controls (C)**: Bilateral Percutaneous Kyphoplasty.

**Outcome (O)**: To evaluate which surgical approach—unilateral or bilateral—offers superior efficacy and safety in the management of osteoporotic vertebral compression fractures.

Inclusion criteria

1. Studies directly comparing unilateral and bilateral percutaneous kyphoplasty in patients with a single acute osteoporotic vertebral compression fracture (OVCF).
2. Adult patients (>40 years) with recent thoracic or lumbar OVCFs.
3. Studies reporting at least one clinical, radiological, perioperative, or safety outcome.
4. Studies including at least 10 patients in each treatment group.
5. Randomized controlled trials (RCTs), prospective comparative studies, or retrospective comparative studies.

Exclusion criteria:

1. Studies including multiple osteoporotic vertebral compression fractures (≥2 OVCFs).
2. Case reports, case series, or studies including fewer than 10 patients per treatment group.
3. High-energy traumatic vertebral fractures or polytrauma patients. Patients with a history of previous surgery at the index vertebral level. Pathological vertebral fractures secondary to primary or metastatic malignancy, infection, or other non-osteoporotic causes.
4. Studies evaluating vertebral augmentation techniques using implantable devices rather than conventional balloon kyphoplasty.
5. Experimental, cadaveric, biomechanical, finite element, animal, or in vitro studies. Review articles, systematic reviews, meta-analyses, narrative reviews, editorials, letters, conference abstracts, technical notes, and expert opinions. Studies with insufficient data for eligibility assessment, methodological quality assessment, or outcome extraction.
6. Studies including chronic OVCFs, fractures of uncertain chronicity, or studies in which recent and chronic fractures could not be analyzed separately.

### 2.2. Assessment of Fracture Acuity

Because definitions of fresh or acute OVCFs vary in the literature [41,42], fracture acuity was additionally assessed across the included studies. For each study, we extracted the reported duration of symptoms or time from injury, the use of MRI, the presence of MRI findings compatible with a recent fracture—including bone marrow edema (BME), T1-weighted hypointensity, T2-weighted hyperintensity, and STIR/fat-suppressed hyperintensity—and other relevant radiological criteria. When MRI was reported as part of the diagnostic work-up but specific imaging criteria for fracture acuity were not provided, this was recorded as such rather than assuming imaging confirmation. The ascertainment of fracture acuity varied across the included studies: several studies used MRI findings consistent with a recent fracture, whereas others used MRI without explicitly defining imaging criteria for acuity or relied predominantly on temporal and clinical criteria. Consequently, no uniform imaging-based definition of fracture acuity was applied across all included primary studies (Supplementary Table S1).

MRI can identify recent OVCFs through characteristic BME-related signal changes, typically including T1-weighted hypointensity and T2/STIR hyperintensity. Although these changes are commonly associated with acute or subacute fractures, their duration is variable and may persist beyond the conventional early post-fracture period, particularly in older patients and in the presence of delayed healing or persistent biomechanical instability [41,42]. Therefore, MRI findings alone cannot provide a uniform chronological definition of fracture age across studies. Considering the variability in temporal, clinical, and imaging criteria used by the included studies, we adopted the term **“recent OVCF”** rather than “acute” or “fresh OVCF” throughout this meta-analysis, based on the combined assessment of the reported clinical history, clinical findings, and radiological data.

### 2.3. Risk of Bias and Methodological Quality Assessment

The risk of bias of the included RCTs was assessed using the Cochrane Risk of Bias 2 (Table 1).

For non-randomized comparative studies, ROBINS-I was used as the primary risk-of-bias instrument, evaluating bias due to confounding, selection of participants, classification of interventions, deviations from intended interventions, missing data, measurement of outcomes, and selection of the reported result (Table 2). The Newcastle–Ottawa Scale (NOS) was additionally applied as a complementary methodological-quality assessment covering study selection, comparability, and outcome ascertainment. Because the two instruments evaluate methodological quality from different perspectives, ROBINS-I was used for the primary risk-of-bias interpretation, whereas NOS was considered supportive and is reported in the Supplementary Material as Supplementary Table S3, ‘Newcastle–Ottawa Scale assessment of the included non-randomized studies.’

For the ROBINS-I domain of bias due to confounding, clinically important potential confounders were defined according to their potential association with both treatment selection and the outcomes of interest. These included ages; baseline BMD/osteoporosis severity; vertebral level; fracture severity and morphology, including degree of vertebral collapse, baseline vertebral height, kyphotic deformity, and posterior-wall involvement where available. Baseline pain and functional status (VAS/ODI or corresponding functional measures) and treatment-selection factors related to fracture asymmetry, vertebral/pedicle anatomy, technical feasibility of unilateral access, and surgeon-related factors were also reported. For each study, the availability and baseline comparability of these factors and the use of statistical methods to control for confounding were considered when assigning the ROBINS-I judgment. Variables occurring after selection of the surgical approach, such as cement volume, were not regarded as universal baseline confounders because they may themselves be influenced by the intervention.

Risk-of-bias assessments were performed independently by two reviewers, with disagreements resolved by discussion and, when necessary, consultation with a third reviewer.

**Selection of Studies**

The search process was blinded, with only article titles and abstracts reviewed initially. Two independent Orthopedic Spine Surgeons used as observers assessed the quality and bias of the retrieved articles. Studies were selected based on the inclusion criteria to minimize bias in both study selection and data extraction.

**Key Steps in the Review Process**

Initial Selection

Articles were evaluated based on titles and abstracts. Full texts were retrieved for studies with unclear inclusion/exclusion criteria.

Resolution of Doubts

If uncertainties persisted, the decision was made through discussion, with a third reviewer brought in if necessary.

### 2.4. Study Protocol and Data Extraction

Before starting the literature review, a written study protocol was developed with clearly defined eligibility criteria. Two independent investigators extracted the relevant data from each search using a standardized form and the same investigators independently extracted the data from each article to ensure consistency. This approach was intended to ensure systematic and consistent study selection and data extraction. The main characteristics of the 11 studies included in the analysis are summarized in Table 3.

Fracture morphology and baseline radiographic characteristics were reported heterogeneously across the included studies (Supplementary Table S2). Fracture location ranged from thoracic and thoracolumbar levels to the lower lumbar spine, with most studies including fractures concentrated around the thoracolumbar junction, whereas Qian et al. [16] evaluated exclusively L3–L5 fractures. Several studies provided quantitative baseline measures of vertebral compression or vertebral height, and baseline kyphotic angles were available in a subset of studies. Where reported separately for the unilateral and bilateral groups, these radiographic parameters were generally comparable at baseline, although Song et al. [45] reported a significantly greater baseline kyphotic angle in the unilateral group (11.96 ± 5.64° vs. 8.60 ± 4.03°, *p* = 0.026).

Fracture morphology was not uniform across studies. Wang et al. [9] restricted inclusion to CT-confirmed AO type A1 fractures, whereas Wan et al. [49] included AO Spine A3/A4 osteoporotic thoracolumbar burst fractures with posterior wall involvement and <25% spinal canal occupancy. Tang et al. [44] and Liu et al. [46] required an intact posterior vertebral wall, while posterior wall status was incompletely reported in several other studies. Qian et al. [16] classified fractures according to wedging, biconcavity, and crush morphology and found no significant difference in morphology distribution between the unilateral and bilateral groups. Intravertebral cleft status was generally not reported. In some studies, fracture asymmetry influenced technical planning of unilateral access, including selection of the puncture side toward the more severely compressed or affected side. Detailed study-level morphological and baseline radiographic characteristics are provided in Supplementary Table S2.

The protocol for this meta-analysis has been registered in the PROSPERO database (Registration No: CRD420261341620).

The data supporting this meta-analysis have been deposited in the Mendeley Data repository and can be accessed at: https://data.mendeley.com/datasets/rj9d7p8v8k/1 (13 July 2026).

### 2.5. Outcomes

The following outcomes were studied: (1) pain (VAS)—short-term and long-term, (2) functional outcome (ODI)—short-term and long-term, (3) surgery duration, (4) PMMA volume, (5) radiation exposure, (6) cement leakage, (7) new adjacent vertebral fractures, (8) complications (excluding cement leakage and adjacent fractures), (9) anterior vertebral body height—short-term and long-term, (10) kyphosis Cobb angle—short-term and long-term, (11) loss of kyphosis reduction, (12) hospital stay, (13) intraoperative blood loss, and (14) cement distribution.

### 2.6. Statistical Methods

All continuous outcomes (e.g., VAS, ODI, operative time, cement volume, vertebral height, kyphosis angle) were measured on identical scales across studies; therefore, the mean difference (MD) was used as the summary measure. For dichotomous outcomes (cement leakage, adjacent fractures, overall complications), the odds ratio (OR) was calculated. For studies with zero events in one arm, a continuity correction of 0.5 was applied to enable effect size estimation. Studies with zero events in both arms did not contribute to the pooled odds ratio. Given the anticipated clinical and methodological diversity among the included studies—particularly the mix of randomized and non-randomized designs—a random-effects model with the restricted maximum-likelihood (REML) estimator for the between-study variance (τ^2^) was chosen a priori for all meta-analyses. The REML estimator was chosen because it provides unbiased estimates of the between-study variance (τ^2^), especially when the number of studies is small, and is the recommended approach for continuous outcomes. This approach provides more conservative confidence intervals than a fixed-effect model and is appropriate when the true effect is expected to vary across studies. Heterogeneity was quantified using the I^2^ statistic and tested with Cochran’s Q, with *p* < 0.10 considered indicative of significant heterogeneity. Pre-specified subgroup analyses were performed according to study design (RCT, retrospective, prospective non-randomized) to explore potential sources of heterogeneity. Because RCTs and observational studies differ fundamentally in susceptibility to selection bias and confounding, study design was considered an a priori clinically relevant source of heterogeneity. RCTs and observational studies were therefore analyzed separately by subgroup. For interpretation, RCT evidence was considered the primary level of inference, whereas observational evidence was considered complementary. Overall estimates combining both study designs were retained as secondary/exploratory summaries and were not used as the principal basis for inference when significant subgroup differences, substantial heterogeneity, or directional discordance between study designs were present.

### 2.7. Interpretation of Subgroup Analyses

Subgroup analyses were pre-specified and considered exploratory. Because the number of studies within some subgroups was small, subgroup interaction tests were interpreted cautiously, and I^2^ values within subgroups were not regarded as definitive evidence of homogeneity. Subgroup results are reported together with the number of studies contributing to each subgroup, and the main conclusions of the meta-analysis are based primarily on the overall estimates and sensitivity analyses rather than on subgroup findings alone.

### 2.8. Missing Standard Deviations

When standard deviations were not reported in the original publications, they were estimated from available *p*-values or confidence intervals where possible. In the VAS meta-analyses (short-term and long-term), standard deviations were unavailable for two studies (Chung 2008 [43] and Wang 2012 [48]), which reported means and sample sizes without measures of dispersion. For these studies, the missing SDs were imputed using the mean of the standard deviations reported by the remaining studies within the corresponding analysis. No other outcome required imputation of missing standard deviations. The influence of these imputations was examined in sensitivity analyses excluding both studies from the VAS meta-analyses; see Supplementary Table S5. Sensitivity analyses for VAS outcomes after excluding studies with imputed standard deviations. Additional sensitivity analyses were conducted by sequentially removing studies identified as influential outliers. Publication bias was evaluated by visual inspection of funnel plots and by Egger’s linear regression test when at least ten studies were available; for analyses with fewer studies, formal testing was omitted in accordance with current recommendations. When Egger’s test suggested asymmetry, the trim-and-fill method was applied to estimate the potential impact of missing studies. All analyses were performed in R using the *meta* and *metafor* packages.

### 2.9. Identification of Influential Studies

For each meta-analysis, influence diagnostics were performed using the following pre-specified criteria: (i) absolute studentized residual > 2.0; (ii) Cook’s distance > 1.0; (iii) a change in the pooled mean difference of more than 0.5 points when the study was omitted in leave-one-out analysis; and (iv) a reduction in I^2^ of at least 40 percentage points after omission of the study. Studies meeting all four criteria were considered influential and were examined further in sensitivity analyses. No study was excluded from the primary meta-analysis on statistical grounds alone.

### 2.10. Certainty of Evidence Assessment

The certainty of the evidence for each pre-specified outcome was evaluated using the GRADE framework. Two reviewers independently assessed the evidence across five domains: risk of bias, inconsistency, indirectness, imprecision, and publication bias. Disagreements were resolved by discussion. The overall certainty was classified as high, moderate, low, or very low. A Summary of Findings (SoF) table was constructed for the following outcomes: short- and long-term pain (VAS), short- and long-term functional status (ODI), cement leakage, new adjacent vertebral fractures, operative time, cement volume, vertebral body height restoration, and kyphosis correction. The SoF table was generated using GRADEpro GDT software (McMaster University and Evidence Prime, Hamilton, ON, Canada, 2026).

## 3. Results

Literature search and selection of studies

**Figure 1:**
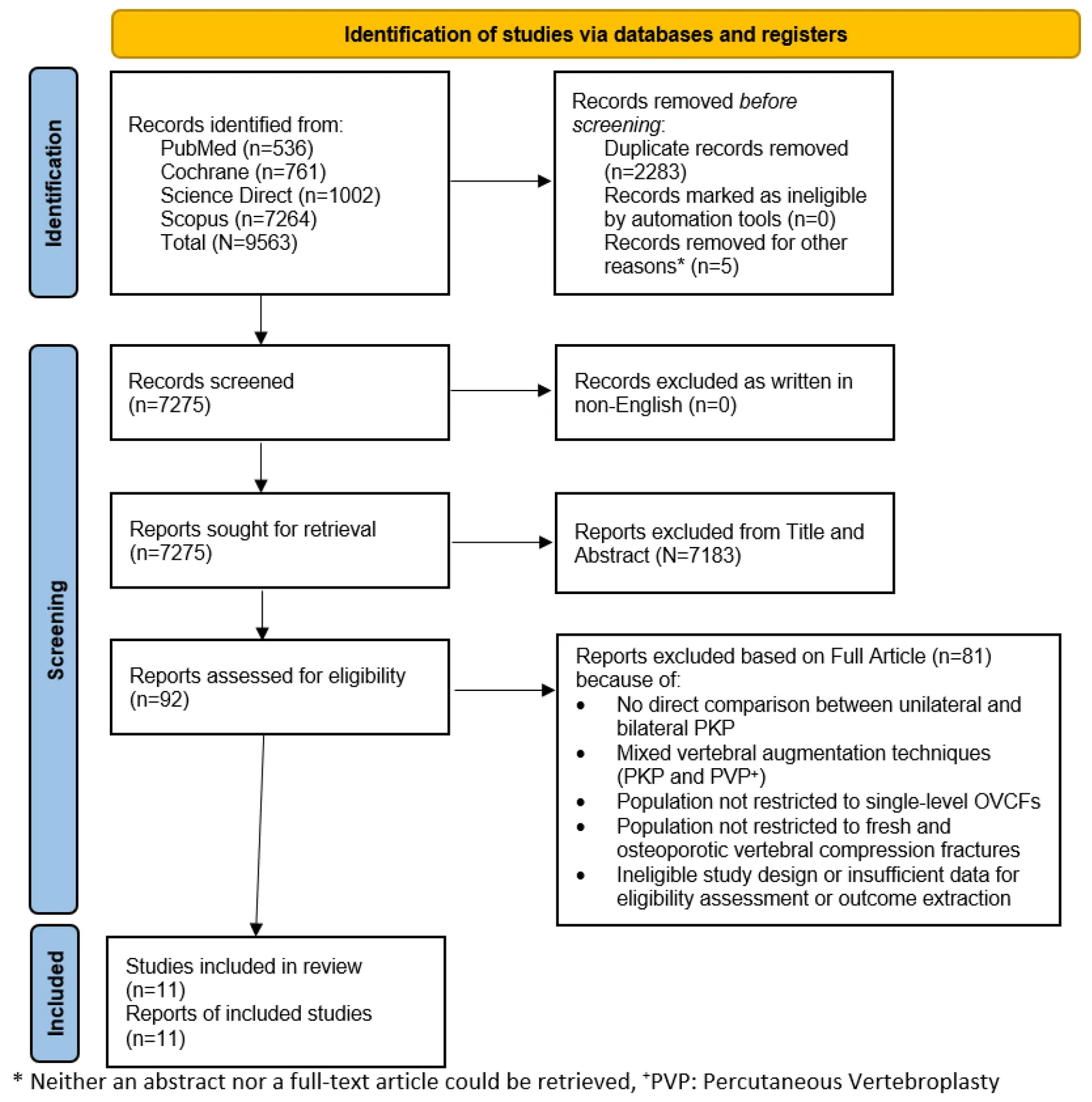
PRISMA 2020 flow diagram.

After the electronic search, 9563 records were identified across four databases (PubMed, Cochrane Library, ScienceDirect, and Scopus). Of these, 2283 duplicate records were removed. Five reports for which neither an abstract nor a full-text article could be retrieved were excluded before eligibility assessment. Of the remaining 7275 records, 7183 were excluded after title and abstract screening. The remaining 92 reports were assessed for eligibility, of which 81 were excluded after full-text assessment. The principal reasons for exclusion were: (1) absence of a direct comparison between unilateral and bilateral PKP; (2) inclusion of mixed vertebral augmentation techniques (PKP and percutaneous vertebroplasty) without separately extractable PKP data; (3) study populations not restricted to single-level OVCFs; (4) study populations not restricted to fresh osteoporotic vertebral compression fractures; and (5) ineligible study design or insufficient data for eligibility assessment or outcome extraction. Finally, 11 studies fulfilled all inclusion criteria and were included in the systematic review and meta-analysis (Figure 1). The included studies were published between 2008 and 2025.

Studies common characteristics

A total of 11 studies, including 1374 patients published between 2000 and 2025, were included in this systematic review and meta-analysis. Study designs comprised seven retrospective studies, one prospective study, and three RCTs. Of the patients, 705 underwent unilateral surgery and 669 bilateral. All included studies reported outcomes for single-level OVCFs (Table 3).

**OVCFs levels** ranged from T6 to L5, most frequently involving the thoraco-lumbar junction (T10-L2). Percutaneous kyphoplasty (PKP) was used in 11 studies and reported preoperative symptom duration ranged from <2 to 3.6 weeks in seven studies.

The **mean patient age** was 70.5 years (range, 44–88 years) in the unilateral PKP group and 71.5 years (range, 48–89 years) in the bilateral PKP group. The **type of anesthesia** was reported in 10 studies: seven studies used local anesthesia, two used general anesthesia, and one study employed a combination of both. Anesthesia type was not specified in one study.

**Hospital cost** was reported in one study as 2.38 ± 0.08 for unilateral versus 2.74 ± 0.07 for bilateral (*p* = 0.021), though currency units were unspecified.

Risk of Bias and Overall Evidence

The three included RCTs were judged as having some concerns overall according to the Cochrane RoB 2 assessment, with no RCT classified as being at high overall risk of bias. Among the eight non-randomized studies, seven were judged to have an overall moderate risk of bias and one (Song et al., 2009 [45]) a serious risk according to ROBINS-I, primarily reflecting limitations related to confounding and treatment selection. The complementary NOS assessment was broadly consistent with acceptable methodological quality among the non-randomized studies (Supplementary Table S3). Overall, the evidence base should therefore be interpreted as comprising a limited number of randomized trials supplemented predominantly by observational evidence with moderate risk of bias.

Summary of Findings and Certainty of Evidence

The overall certainty of evidence, as assessed by the GRADE framework, is summarized in Table 4. No outcome was rated as high certainty, primarily because the body of evidence included observational studies and, for several outcomes, substantial heterogeneity was observed.

- **Moderate certainty** was assigned to cement leakage (I^2^ = 0%) and new adjacent vertebral fractures (I^2^ = 0%), reflecting consistent findings across study designs, with downgrading for the inclusion of non-randomized studies.
- **Low certainty** was assigned to short- and long-term pain (VAS) and functional status (ODI). For short-term VAS, the presence of significant heterogeneity (I^2^ = 75.5%) and divergent findings between RCTs and retrospective studies contributed to the downgrade.
- **Very low certainty** was assigned to operative time, cement volume, vertebral height restoration, and kyphosis correction, mainly due to very serious inconsistency (I^2^ > 90%) and/or imprecision from small sample sizes. These ratings reinforce the interpretation that, although several differences reached nominal statistical significance, the confidence in the magnitude and consistency of these estimates is limited.

Meta-Analysis Functional Outcomes.

### 3.1. Pain (VAS)—Short-Term

**Figure 2:**
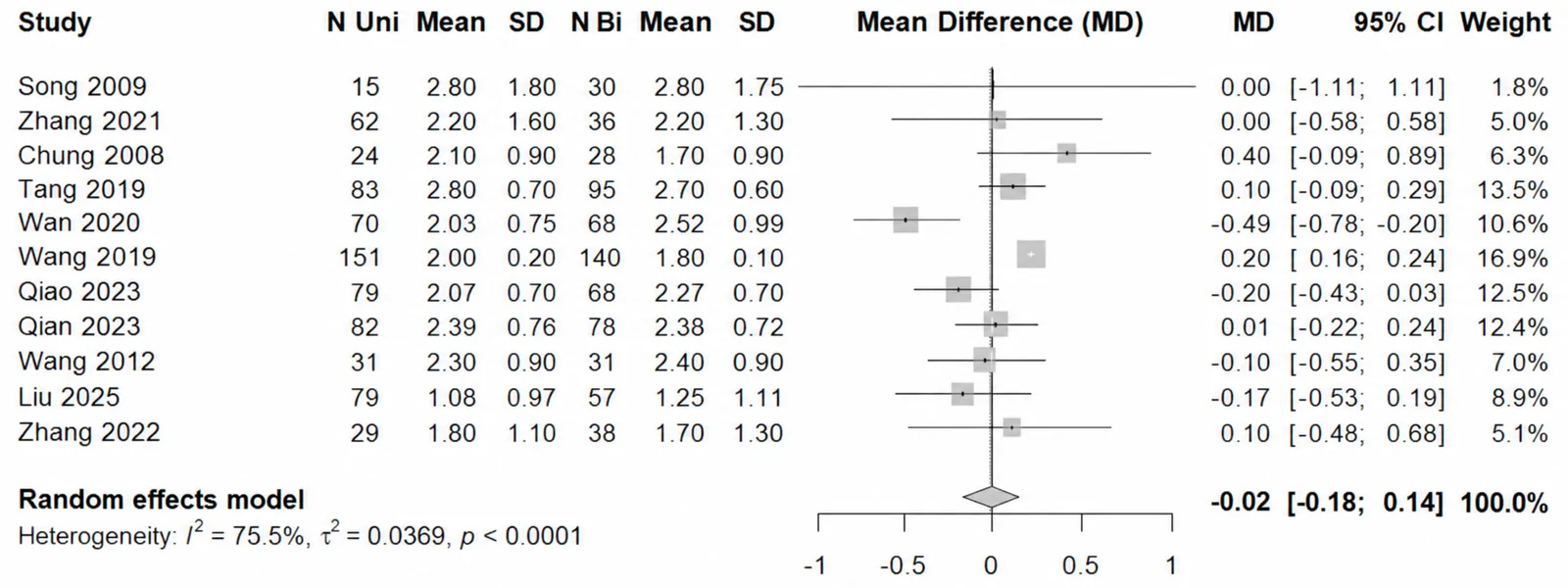
Forest plot comparing short-term postoperative VAS scores between unilateral and bilateral percutaneous kyphoplasty [9,15,16,43,44,45,46,47,48,49,50].

Eleven studies (n = 1374) reported short-term postoperative VAS scores (≤3 months). Under a random-effects model, the mean difference between unilateral and bilateral PKP was −0.02 points (95% CI: −0.18 to 0.14; *p* = 0.80; Figure 2).

Substantial between-study heterogeneity was observed (I^2^ = 75.5%, *p* < 0.001). In subgroup analysis by study design, the three RCTs (k = 3) showed low inconsistency (I^2^ = 0%) and indicated a small but statistically significant advantage of bilateral PKP (MD = 0.20, 95% CI: 0.16 to 0.23; *p* < 0.001). The seven retrospective studies (k = 7) showed low inconsistency (I^2^ = 20.1%) but yielded an effect in the opposite direction, favoring unilateral PKP (MD = −0.17, 95% CI: −0.33 to −0.01; *p* = 0.04). The single prospective non-randomized study (k = 1) showed no statistically significant difference. The test for subgroup differences was statistically significant (*p* < 0.001), indicating a clear divergence in effect estimates according to study design. However, given the limited number of studies, particularly RCTs, this finding should be interpreted cautiously.

When the two studies with imputed standard deviations (Chung 2008 [43] and Wang 2012) [48] were excluded, the pooled MD for short-term VAS was −0.045 points (95% CI: −0.215 to 0.125; *p* = 0.602; I^2^ = 79.3%), compared with −0.02 points (95% CI: −0.18 to 0.14; *p* = 0.80; I^2^ = 75.5%) in the primary analysis. Thus, exclusion of the studies with imputed standard deviations did not materially alter the overall estimate or its interpretation.

Funnel plot inspection and Egger’s test suggested possible asymmetry for short-term VAS (*p* = 0.049). Trim-and-fill analysis added five hypothetical studies and shifted the pooled MD from −0.02 to 0.18 points (95% CI: 0.01 to 0.36; *p* = 0.04). However, these findings should be interpreted with caution for two reasons. First, substantial between-study heterogeneity was present (I^2^ = 75.5%), which may itself contribute to funnel plot asymmetry in the absence of publication bias. Second, the divergent results of randomized and retrospective studies represent one possible explanation for the observed asymmetry, although publication bias cannot be definitively excluded. The adjusted estimate produced by the trim-and-fill method should therefore not be regarded as definitive.

Overall, short-term pain outcomes showed a clear divergence between randomized and retrospective evidence. The RCTs favored bilateral PKP, whereas the retrospective studies favored unilateral PKP, although the absolute effect estimates were small in both subgroups. Given this discordance and the potential for residual confounding and treatment-selection effects in observational studies, the combined estimate should not be regarded as the primary measure of comparative efficacy. Greater inferential weight should therefore be given to the randomized evidence, while the observational findings should be considered complementary.

### 3.2. Pain (VAS)—Long-Term

**Figure 3:**
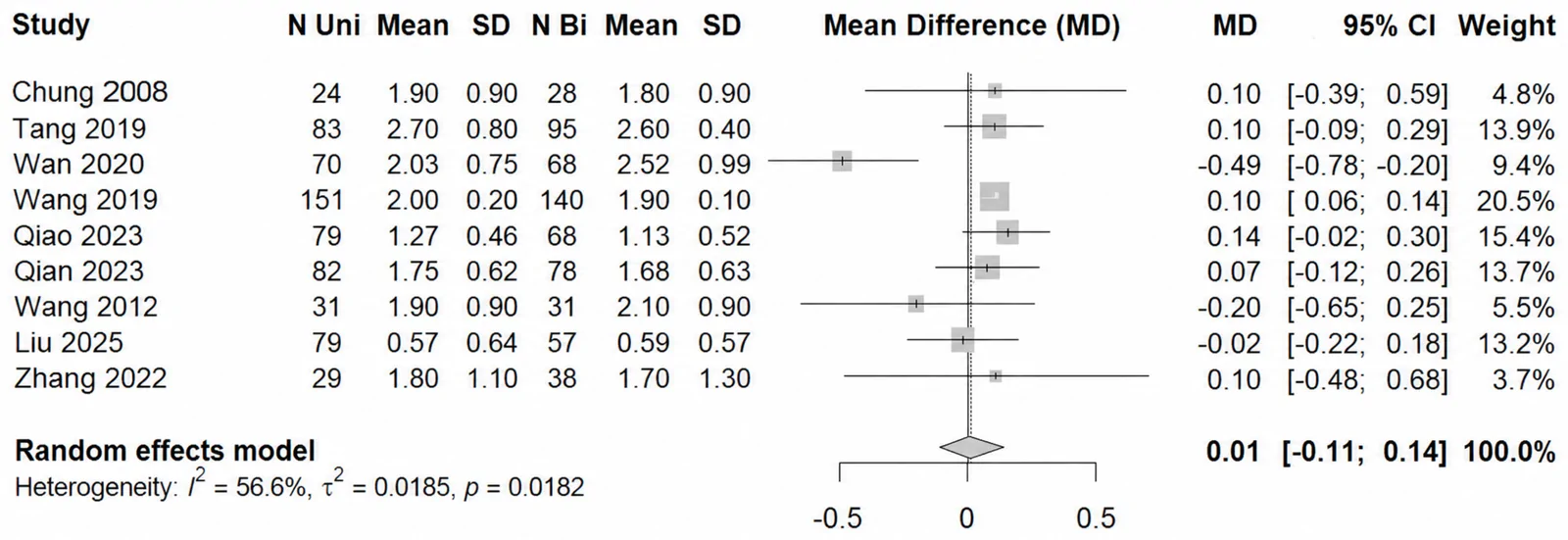
Forest plot comparing long-term postoperative VAS scores between unilateral and bilateral percutaneous kyphoplasty [9,15,16,43,44,46,48,49,50].

Nine studies (n = 1231) reported long-term VAS scores (≥6 months). The pooled MD was 0.01 points (95% CI: −0.11 to 0.14; *p* = 0.82; I^2^ = 56.6%; Figure 3).

Heterogeneity was moderate and substantially lower than in the short-term analysis. In the long-term analysis, the three RCTs (k = 3) showed low inconsistency (I^2^ = 0%) with an MD of 0.10 (95% CI: 0.06 to 0.14; *p* < 0.001). The five retrospective studies (k = 5) showed moderate to substantial inconsistency (I^2^ = 73.2%) and a non-significant difference in the opposite direction (MD = −0.07, 95% CI: −0.29 to 0.15; *p* = 0.53). The test for subgroup differences was not significant (*p* = 0.32), suggesting that, in the long term, study design does not strongly influence the estimated effect. However, because the number of studies in each subgroup was limited, this convergence should be interpreted cautiously. Egger’s test was not significant (*p* = 0.41), and the trim-and-fill procedure added only one hypothetical study without materially altering the pooled estimate. Overall, no statistically significant long-term difference in pain outcomes was identified between the two approaches.

When the two studies with imputed standard deviations (Chung 2008 [43] and Wang 2012 [48]) were excluded, the pooled MD for long-term VAS was 0.020 points (95% CI: −0.118 to 0.157; *p* = 0.781; I^2^ = 64.3%). This result was consistent with the primary analysis (MD = 0.01, 95% CI: −0.11 to 0.14; *p* = 0.82), indicating that exclusion of the studies with imputed standard deviations did not materially alter the interpretation.

In conclusion, long-term pain outcomes showed greater convergence between randomized and retrospective evidence than short-term outcomes. Although the subgroup point estimates remained in opposite directions, the test for subgroup differences was not statistically significant. Nevertheless, given the limited number of studies and the residual heterogeneity among retrospective studies, these findings should be interpreted cautiously. Overall, the available evidence does not demonstrate a statistically significant long-term difference in pain outcomes between unilateral and bilateral PKP.

### 3.3. Oswestry Disability Index (ODI)—Short-Term

Six studies (n = 959) reported short-term postoperative ODI scores. In the primary meta-analysis including all eligible studies, the pooled MD was 0.84 points (95% CI: 0.06 to 1.61; *p* = 0.034; I^2^ = 87.8%), indicating a statistically significant but clinically negligible difference favoring the bilateral approach. However, extreme between-study heterogeneity was present (*p* < 0.001).

**Figure 4:**
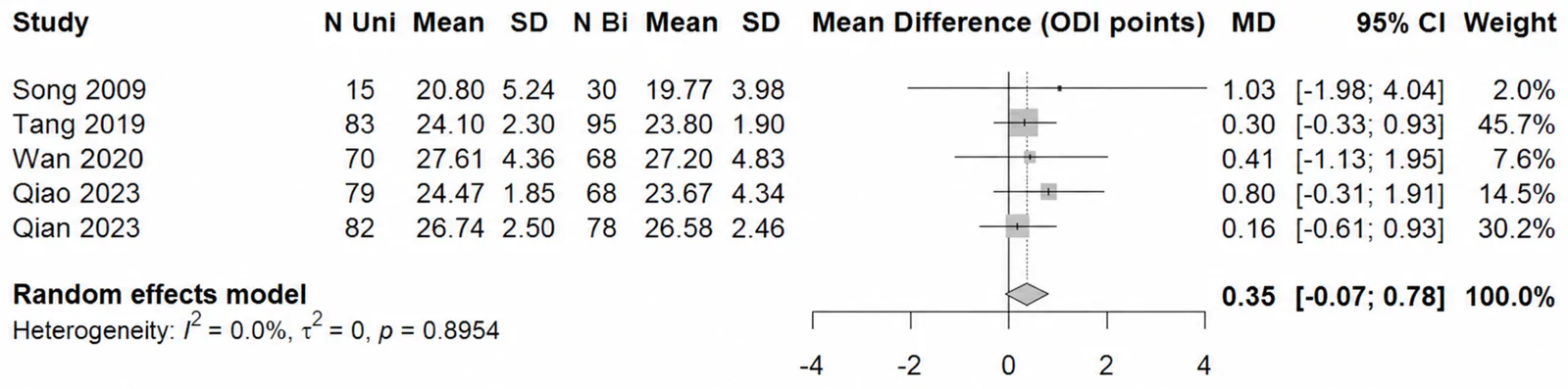
Forest plot comparing short-term postoperative ODI scores between unilateral and bilateral percutaneous kyphoplasty [15,16,44,45,49].

Influence diagnostics identified Wang 2019 [9] as an influential study: studentized residual > 2.0, Cook’s distance > 1.0, and omission of the study changed the pooled MD by >0.5 points and reduced I^2^ by more than 40 percentage points. In a sensitivity analysis excluding this study, the pooled MD was 0.35 points (95% CI: −0.07 to 0.78; *p* = 0.102; I^2^ = 0%; Figure 4).

Subgroup analysis of the primary model showed that the four retrospective studies yielded an MD of 0.40 (95% CI: −0.18 to 0.97; I^2^ = 0%), while the two RCTs showed inconsistent results (I^2^ = 95.9%). After excluding the influential study, the remaining RCT (Tang 2019 [44]) reported an MD of 0.30 (95% CI: −0.33 to 0.93).

Although the primary analysis reached nominal statistical significance, the extreme heterogeneity and the sensitivity analysis indicate that the difference is not robust and should be interpreted with caution.

### 3.4. ODI—Long-Term

Five studies (n = 914) provided long-term ODI scores. In the primary meta-analysis including all eligible studies, the pooled MD was 0.93 points (95% CI: 0.18 to 1.68; *p* = 0.014; I^2^ = 93.4%), again favoring the bilateral technique with extreme heterogeneity.

**Figure 5:**
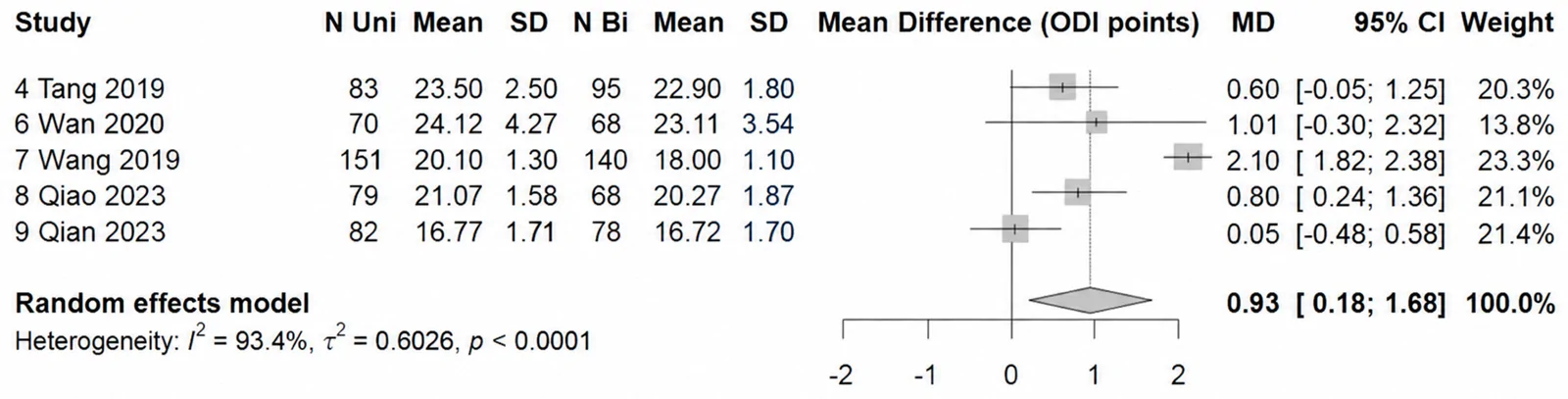
Forest plot comparing long-term postoperative ODI scores between unilateral and bilateral percutaneous kyphoplasty [9,15,16,44,49].

Influence diagnostics identified the same study (Wang 2019 [9]) as influential. In a sensitivity analysis excluding this study, heterogeneity decreased to an acceptable level (I^2^ = 33.9%) and the pooled MD was 0.52 points (95% CI: 0.09 to 0.95; *p* = 0.018; k = 4; Figure 5).

Subgroup analysis of the sensitivity model showed consistent results across study designs (test for subgroup differences *p* = 0.85): the single remaining RCT (Tang 2019 [44]) reported an MD of 0.60 (95% CI: −0.05 to 1.25), and the three retrospective studies yielded a pooled MD of 0.51 (95% CI: −0.09 to 1.12; I^2^ = 54.3%).

Although statistically significant, the between-group difference was only 0.52 points on the 100-point ODI scale, substantially below the published MCID thresholds of 12.8 points for ODI [51].

Meta-Analysis Perioperative Parameters

### 3.5. Surgery Duration

Nine studies (n = 1374) provided data on operative time. The overall random-effects meta-analysis showed a statistically significant reduction of approximately 10 min with unilateral PKP (MD = −10.2 min, 95% CI: −16.6 to −3.8; *p* = 0.002). However, between-study heterogeneity was extreme (I^2^ = 98.5%, *p* < 0.001), substantially limiting the interpretability of the overall pooled estimate. Subgroup analysis by study design revealed a statistically significant interaction (*p* = 0.0003). The six retrospective studies (k = 6) suggested a larger reduction in operative time with unilateral PKP (MD = −15.2 min, 95% CI: −21.2 to −9.2; I^2^ = 95.6%), whereas the two RCTs (k = 2) showed no statistically significant between-group difference (MD = +1.2 min, 95% CI: −4.5 to +6.8; *p* = 0.69; I^2^ = 96.9%). The single prospective non-randomized study (k = 1) reported an intermediate difference. However, the very small number of RCTs and the substantial within-subgroup inconsistency limit the strength of this subgroup comparison, which should therefore be considered exploratory.

To further explore the extreme heterogeneity, leave-one-out sensitivity analyses were performed. Removal of Wang 2018 changed the pooled MD to −12.0 min (95% CI: −18.0 to −6.0), whereas removal of Qiao 2023 [15] changed it to −8.2 min (95% CI: −13.9 to −2.4) [15,48]. Excluding both Qiao 2023 and Wang 2012 reduced the pooled MD to −6.6 min (95% CI: −12.0 to −1.3), although heterogeneity remained extreme (I^2^ = 97.7%) [15,48]. These analyses indicate that no single study fully accounted for the observed heterogeneity and that the magnitude of the overall pooled effect was sensitive to the contribution of individual retrospective studies.

Overall, the pooled estimate for operative time should be interpreted cautiously because of the extreme heterogeneity and the divergence between randomized and observational evidence. Although the two RCTs showed no statistically significant difference in operative time (MD = +1.2 min, 95% CI: −4.5 to +6.8), the limited number of randomized studies and the substantial inconsistency between them preclude a definitive conclusion regarding the magnitude of any between-group difference.

### 3.6. Injected PMMA Volume per Vertebra

Seven studies (n = 1117) reported the volume of cement injected per vertebra. The overall random-effects meta-analysis showed a statistically significant reduction in cement volume with unilateral PKP (MD = −1.31 mL, 95% CI: −2.18 to −0.44; *p* = 0.003), although between-study heterogeneity was substantial (I^2^ = 96.0%). Subgroup analysis according to study design yielded different estimates: the two RCTs (k = 2) showed low inconsistency (I^2^ = 0%) and a pooled MD of −0.49 mL (95% CI: −0.58 to −0.40; *p* < 0.001), whereas the five retrospective studies (k = 5) reported a substantially larger and more variable reduction with unilateral PKP (MD = −1.96 mL; I^2^ = 94.2%; *p* < 0.001). The test for subgroup differences was statistically significant (*p* = 0.001). However, given the small number of RCTs and the substantial heterogeneity among retrospective studies, this subgroup comparison should be regarded as exploratory rather than definitive.

Sensitivity analyses excluding the two most extreme retrospective studies (Wang 2012 and Zhang 2021) [47,48] progressively reduced the magnitude of the overall MD to −0.78 mL and, after exclusion of all retrospective studies, to −0.33 mL. These findings indicate that the magnitude of the overall pooled effect was influenced by the observational evidence. Overall, the randomized evidence indicates that unilateral PKP uses approximately 0.5 mL less cement than bilateral PKP. Although this difference was statistically significant, its clinical relevance remains uncertain.

### 3.7. Radiation Exposure

Five studies (n = 765) quantified intraoperative fluoroscopy shots. The pooled MD under a random-effects model was −7.97 shots (95% CI: −17.44 to +1.50; *p* = 0.099; I^2^ = 97.9%). The two RCTs reported opposite and non-significant findings (Tang 2019) [44]: −1.9 shots; Wang 2018 [9]: +1.3 shots; pooled MD = −0.29, *p* = 0.86), while retrospective studies varied enormously (range: −4.8 to −28 shots). Removal of the most influential studies did not stabilize the estimate.

The available data are too heterogeneous to provide a reliable estimate of the effect on radiation exposure. The highest-quality evidence from RCTs suggests no clinically meaningful difference.

### 3.8. Cement Leakage

Eight studies recorded cement leakage events. One RCT (Tang 2019) [44] had zero events in both arms and did not contribute to the quantitative synthesis. The remaining seven studies (n = 1073; 152 events) showed low inconsistency (I^2^ = 0%). The pooled odds ratio was 1.01 (95% CI: 0.71–1.45; *p* = 0.94), indicating no statistically significant difference in the risk of cement leakage between unilateral and bilateral PKP. Subgroup analysis showed a non-significant difference between the two RCTs (k = 2; OR = 1.32, 95% CI: 0.38–4.60; I^2^ = 23.8%) and the five retrospective studies (k = 5; OR = 0.97, 95% CI: 0.60–1.57; I^2^ = 0%). The test for subgroup differences was not statistically significant (*p* = 0.65). However, the small number of studies in the RCT subgroup limits the ability to detect a true subgroup difference.

Overall, the available evidence did not demonstrate a statistically significant difference in cement leakage risk between unilateral and bilateral PKP, and the pooled estimate showed little between-study inconsistency. Nevertheless, the limited number of randomized studies warrants cautious interpretation of the comparative safety evidence.

### 3.9. New Adjacent Vertebral Fractures

Eight studies (n = 1157) provided data on subsequent adjacent vertebral fractures. One study (Qian 2023) [16] recorded no events in either group and did not contribute to the quantitative synthesis. The seven evaluable studies, comprising 79 events, showed low inconsistency (I^2^ = 0%). The pooled OR was 0.97 (95% CI: 0.61–1.55; *p* = 0.90), indicating no statistically significant difference in the risk of new adjacent vertebral fractures between unilateral and bilateral PKP. In subgroup analysis, the two RCTs (k = 2) yielded an OR of 1.02 (95% CI: 0.56–1.88; I^2^ = 0%), whereas the five retrospective studies (k = 5) yielded an OR of 0.90 (95% CI: 0.43–1.87; I^2^ = 0%). The test for subgroup differences was not statistically significant (*p* = 0.79). However, the limited number of studies in each subgroup restricts the ability to detect a potentially relevant subgroup effect.

Overall, the available evidence did not demonstrate a statistically significant difference in the risk of new adjacent vertebral fractures between unilateral and bilateral PKP. Although the findings were consistent across study designs and showed little between-study inconsistency, the limited number of studies and the relatively wide confidence intervals warrant cautious interpretation.

### 3.10. Complications (Excluding Cement Leakage and Adjacent Fractures)

Eleven studies were evaluated for other procedure-related complications, of which seven reported no events. Among the four informative studies (80 events), overall heterogeneity was substantial (I^2^ = 84.2%). Subgroup analysis suggested a divergence between the three retrospective studies (k = 3; OR = 0.43, 95% CI: 0.14–1.32) and the single RCT with events (k = 1; OR = 3.49, 95% CI: 1.44–8.48). The test for subgroup differences was statistically significant (*p* = 0.004). However, because the randomized subgroup comprised only one informative study, this comparison should be regarded as exploratory and interpreted with considerable caution.

Overall, the available evidence was conflicting and insufficient to determine whether unilateral and bilateral PKP differ in their overall complication profiles. The higher complication rate observed with unilateral PKP in the single informative RCT (Wang 2018) [9] was primarily driven by nerve root stimulation; however, this finding requires confirmation in additional randomized studies and should not be generalized to the overall safety of unilateral PKP.

Furthermore, because the pooled endpoint comprised clinically heterogeneous adverse events, individual procedure-related complications are presented separately by study and treatment arm in Supplementary Table S4. These included, where reported, nerve root irritation/stimulation, neurological events, infection, bleeding/hematoma, pedicle injury, pulmonary cement embolism, and other procedure-related adverse events.

### 3.11. Anterior Vertebral Body Height—Short-Term

Four studies (n = 530) reported short-term (≤3 months) postoperative anterior vertebral height. The overall analysis showed low inconsistency (I^2^ = 0%) and a statistically significant difference of approximately 0.5 mm favoring bilateral PKP (MD = −0.51 mm, 95% CI: −0.88 to −0.15; *p* = 0.006). In subgroup analysis, the single RCT (k = 1) reported an MD of −0.60 mm, the two retrospective studies (k = 2) yielded an MD of −0.47 mm (I^2^ = 0%), and the single prospective non-randomized study (k = 1) reported an MD of −0.50 mm. The test for subgroup differences was not statistically significant (*p* = 0.95). However, the very small number of studies within each subgroup limits the strength of this comparison.

Overall, the available evidence suggests a small radiographic advantage of bilateral PKP in short-term anterior vertebral height restoration. Although the observed difference of approximately 0.5 mm was statistically significant, its clinical relevance remains uncertain.

### 3.12. Anterior Vertebral Body Height—Long-Term

Three studies (n = 392) provided long-term (≥6 months) anterior vertebral height measurements. The pooled analysis showed low inconsistency (I^2^ = 0%) and a small statistically significant difference favoring bilateral PKP (MD = −0.44 mm, 95% CI: −0.88 to −0.005; *p* = 0.048). In subgroup analysis, the single RCT (Tang 2019 [44]) reported an MD of −0.60 mm, the single retrospective study (Qiao 2023 [15]) an MD of −0.30 mm, and the single prospective non-randomized study (Zhang 2022 [50]) an MD of −0.50 mm. The test for subgroup differences was not statistically significant (*p* = 0.82). However, with only one study in each subgroup, no firm conclusions regarding differences according to study design can be drawn.

Overall, the available evidence suggests that the small radiographic advantage of bilateral PKP in vertebral height restoration may persist at long-term follow-up. However, the magnitude of the difference was small, the statistical significance was borderline, and its clinical relevance remains uncertain.

### 3.13. Kyphosis Cobb Angle—Short-Term

Four studies (n = 754) assessed short-term kyphosis correction. Overall heterogeneity was substantial (I^2^ = 74.5%). In exploratory subgroup analysis, the two RCTs (k = 2) showed low inconsistency (I^2^ = 0%) and favored the bilateral approach (MD = 0.76°, 95% CI: 0.32° to 1.20°; *p* < 0.001), whereas the two retrospective studies (k = 2) showed a non-significant effect in the opposite direction, favoring the unilateral approach (MD = −0.36°, 95% CI: −0.83° to 0.11°; *p* = 0.13; I^2^ = 0%). The test for subgroup differences was statistically significant (*p* < 0.001). However, with only two studies in each subgroup, these findings should be interpreted cautiously and cannot establish that study design fully accounts for the observed heterogeneity.

Overall, randomized and retrospective evidence showed divergent effect estimates for short-term kyphosis correction. The RCTs demonstrated a statistically significant advantage of approximately 0.8° with bilateral PKP, whereas the retrospective studies showed no statistically significant difference. Given the limited number of studies, greater inferential weight should be given to the randomized evidence; however, the magnitude of the observed radiographic difference was small, and its clinical relevance remains uncertain.

### 3.14. Kyphosis Cobb Angle—Long-Term

Three studies (n = 616) provided long-term angular measurements. The overall analysis showed substantial between-study heterogeneity (I^2^ = 96.0%). In subgroup analysis, the two RCTs (k = 2) indicated a statistically significant advantage of bilateral PKP (MD = 1.58°, 95% CI: 0.12° to 3.04°; *p* = 0.034), although substantial inconsistency remained within the randomized subgroup (I^2^ = 88.9%). The single retrospective study (k = 1) showed no statistically significant difference (MD = −0.29°, 95% CI: −0.79° to 0.21°). The test for subgroup differences was statistically significant (*p* = 0.017). However, because the retrospective subgroup comprised only one study and substantial heterogeneity persisted between the RCTs, this subgroup analysis should be regarded as highly exploratory and not definitive.

Overall, the randomized evidence suggests that bilateral PKP may provide approximately 1–2° greater long-term kyphosis correction. However, given the limited number of RCTs, the substantial inconsistency between them, and the small magnitude of the observed radiographic difference, its clinical relevance remains uncertain.

### 3.15. Loss of Kyphosis Reduction

Only two RCTs reported the loss of kyphosis correction over time, using incompatible metrics (percentage in Chung 2008 [43] vs. absolute degrees in Wang 2018 [9]) and could not be combined. Chung 2008 [43] found a significantly smaller percentage loss in the bilateral group (4.7 ± 2.9% vs. 1.6 ± 1.1%; *p* = 0.0001), while Wang 2018 [9] reported no significant difference in absolute loss (5.35 ± 2.05 vs. 4.87 ± 1.87; *p* = 0.26).

The limited and incompatible data preclude a conclusion on the relative ability of the two approaches to maintain kyphosis correction.

### 3.16. Hospital Stay, Intraoperative Blood Loss and Cement Distribution

Data on hospital stay and intraoperative blood loss were reported in only one or two studies each and could not be meta-analyzed. The available single-study estimates (Qian 2023 [16] for hospital stay, Wan 2020 [49] for blood loss) showed no significant or clinically meaningful differences between groups. Cement distribution was reported quantitatively in two studies using non-comparable metrics; both suggested more homogeneous cement spread with the bilateral approach in the coronal plane, but the evidence is too sparse for definitive conclusions.

Overall, study design influenced the direction and/or magnitude of the estimated treatment effect for several outcomes. The most notable discrepancies between randomized and retrospective evidence were observed for short-term pain relief, operative time, cement volume, and kyphosis correction. In contrast, randomized and observational evidence was more consistent for outcomes such as long-term pain and functional outcomes and cement leakage. These design-related differences should be considered when interpreting overall pooled estimates across study designs.

## 4. Discussion

OVCFs most commonly involve the thoracolumbar spine and represent an important cause of pain, disability, and morbidity in the elderly [52]. In patients with persistent symptoms despite conservative treatment, PKP is an established minimally invasive treatment aimed at stabilizing the fractured vertebra, reducing pain, and partially restoring vertebral height and sagittal alignment [8,53,54,55]. PKP may be performed through either a unilateral or bilateral transpedicular approach, each with potential technical advantages and disadvantages.

The present meta-analysis included 11 studies comprising 1374 patients and specifically focused on fresh, single-level thoracolumbar OVCFs. The principal finding is not simply that unilateral and bilateral PKP produce broadly comparable outcomes, but that study design materially influenced the estimated treatment effect for several outcomes. Directional or magnitude discrepancies between randomized and observational evidence were particularly evident for short-term pain, operative time, cement volume, and kyphosis correction. Accordingly, randomized evidence was considered the primary level of inference when such discrepancies occurred, whereas observational evidence was interpreted as complementary.

Importantly, the divergence between RCT and non-RCT evidence may not represent statistical heterogeneity alone. In routine clinical practice, selection of unilateral or bilateral access is not necessarily random. Surgeons may preferentially select an approach according to vertebral morphology, fracture asymmetry and severity, pedicle anatomy, technical feasibility, or their own experience and preference. More complex fractures may be more likely to receive bilateral access, whereas technically favorable fractures may be treated unilaterally. Several of these variables were incompletely reported or controlled in the retrospective studies included in this review. Consequently, confounding by indication and surgeon selection may partly explain why observational studies sometimes appear to favor unilateral PKP more strongly than randomized trials. This discrepancy is particularly relevant because restriction to fresh, single-level fractures reduces some sources of clinical heterogeneity but cannot eliminate differences in fracture morphology or treatment selection.

### 4.1. Perioperative Outcomes

For operative time, the overall pooled estimate suggested an approximately 10 min advantage for unilateral PKP, but this finding was accompanied by extreme heterogeneity (I^2^ = 98.5%) and was predominantly driven by observational studies. In contrast, the two RCTs showed no statistically significant difference between approaches. Thus, the apparent procedural-time advantage of unilateral PKP in retrospective series should be interpreted cautiously and may partly reflect case selection, surgeon experience, or differences in procedural complexity rather than the pedicular approach alone.

Unilateral PKP required statistically less PMMA cement, consistent with previous reports [7,22,23,24,25,26,28,29,31,32,33,36,38,56,57]. However, randomized evidence indicated an absolute difference of only approximately 0.5 mL relative to typical injection volumes of 3–7 mL. The larger differences observed in retrospective cohorts may again reflect differences in fracture morphology, vertebral size, procedural strategy, or treatment selection. Therefore, although lower cement volume is a reproducible technical feature of unilateral PKP, the clinical relevance of the small difference demonstrated by randomized evidence remains uncertain.

For radiation exposure, substantial heterogeneity precluded a reliable overall estimate. Previous studies have also produced inconsistent findings [7,25,27,32,33,34,36]. The available randomized evidence did not identify a clear between-group difference, suggesting that radiation exposure may depend substantially on procedural technique, surgeon experience, and fluoroscopic practice rather than solely on unilateral versus bilateral access.

### 4.2. Complications

Cement leakage was one of the more consistent findings. No statistically significant difference was identified between unilateral and bilateral PKP, supporting previous meta-analytic evidence reporting similar leakage rates between approaches [7,22,24,25,28,29,30,31,33,34,36]. Similarly, no statistically significant difference was identified in the incidence of adjacent vertebral fractures.

The pooled analysis of other procedure-related complications should be interpreted cautiously because it combines adverse events with different mechanisms and clinical consequences. Individual complications are therefore presented separately in Supplementary Table S4. Notably, Wang et al. [9] reported a higher incidence of nerve root stimulation with unilateral PKP. A possible technical explanation is the greater medial angulation required to achieve adequate contralateral cement distribution through a unilateral approach, potentially increasing the risk of medial pedicle-wall violation and nerve root irritation. However, this finding originated from a single study and should not be generalized as evidence of inferior overall safety of unilateral PKP without corroboration from additional randomized evidence.

### 4.3. Radiological Outcomes

Bilateral PKP achieved statistically greater vertebral height restoration, but the absolute between-group difference was small, approximately 0.5 mm. This difference persisted during follow-up; however, in the absence of an established clinically important threshold for vertebral height restoration after PKP, its clinical relevance remains uncertain.

Moreover, such small local radiographic differences should not be interpreted in isolation from global spinal alignment. Sagittal balance reflects the interaction between spinal alignment, pelvic orientation, and compensatory mechanisms involving the pelvis and lower extremities. In patients undergoing surgery for adult spinal deformity, Perna et al. demonstrated significant associations between global spinopelvic-femoral parameters, particularly T1 pelvic angle (TPA) and femoral obliquity angle (FOA), and postoperative disability and quality of life [58]. Although these findings derive from a different spinal population and cannot be directly extrapolated to patients undergoing PKP, they illustrate the importance of considering global alignment when interpreting small changes in local vertebral morphology.

A study-design discrepancy was again evident for kyphosis correction. Randomized evidence favored bilateral PKP by approximately 0.8° in the short term and suggested a 1–2° advantage during longer follow-up, whereas retrospective evidence did not consistently reproduce this effect. These findings suggest that bilateral access may provide a modest radiographic advantage in restoring local kyphotic alignment. However, the clinical significance of these small angular differences remains uncertain, particularly because the included studies predominantly assessed local or segmental radiographic parameters rather than global sagittal alignment. Statistically detectable differences in local kyphosis therefore cannot be assumed to translate into meaningful differences in global spinopelvic balance, compensatory mechanisms, function, or quality of life.

Evidence regarding loss of kyphosis correction remains insufficient. Only two RCTs reported this outcome, using incompatible metrics: Chung et al. [43] reported percentage loss, whereas Wang et al. [9] reported absolute angular loss. Quantitative pooling was therefore not possible, and no firm conclusion regarding the relative ability of either technique to maintain sagittal correction over time can be drawn.

From a clinical perspective, the small radiographic advantages observed with bilateral PKP raise the possibility that this approach may be more relevant in selected fractures with greater vertebral collapse, more pronounced local kyphotic deformity, or morphology requiring more symmetric vertebral body restoration and cement distribution. Conversely, unilateral access may remain sufficient in less severe or anatomically favorable fractures. However, the available studies did not report fracture morphology and baseline deformity with sufficient consistency to permit subgroup analyses according to collapse severity or kyphosis magnitude. Therefore, these considerations should be regarded as clinically plausible hypotheses rather than evidence-based indications for selecting one approach over the other. Future studies should specifically evaluate whether baseline fracture morphology modifies the comparative effect of unilateral and bilateral PKP.

### 4.4. Functional Outcomes

The clearest study-design discrepancy was observed for short-term pain relief. The three RCTs were internally consistent and demonstrated a small statistically significant difference favoring bilateral PKP, whereas the retrospective studies consistently favored unilateral treatment. The opposing direction of effect despite low within-subgroup heterogeneity strongly suggests that methodological rather than random variation may contribute to this finding. Non-random treatment selection, differences in fracture morphology or severity, and surgeon-related factors are plausible explanations.

This discrepancy was no longer evident at mid- and long-term follow-up, where randomized and observational estimates converged and subgroup differences were not statistically significant. Thus, although the short-term VAS findings are sensitive to study design, no statistically significant long-term difference in pain outcomes was identified between unilateral and bilateral PKP.

For ODI, short-term outcomes did not differ significantly between approaches after exclusion of the influential Wang 2018 study [9]. At long-term follow-up, the pooled difference was statistically significant (MD = 0.52 points, 95% CI 0.09–0.95), but its magnitude was substantially below the published MCID of 12.8 points on the 100-point ODI scale [9]. Thus, the statistical difference should be interpreted in the context of its very small absolute magnitude.

Finally, evidence regarding hospital stay, intraoperative blood loss, and cement distribution was insufficient for quantitative synthesis. Available single-study data did not demonstrate clear differences in hospital stay or blood loss, while studies assessing cement distribution used incompatible measurement methods. In unilateral PKP, cement distribution may additionally depend on technical factors such as puncture trajectory and angulation, which determine the ability to achieve adequate contralateral cement spread. However, these technical variables were inconsistently reported across studies, preventing assessment of their influence on comparative outcomes.

### 4.5. Future Research

The present review highlights the importance of standardized and longitudinal data collection in future comparative studies of vertebral augmentation. Heterogeneous outcome definitions, inconsistent follow-up intervals, and incomplete reporting limited quantitative synthesis for several outcomes. Emerging digital infrastructures, including interoperable orthopedic registries and secure longitudinal data-management systems, may facilitate more standardized collection and traceability of clinical and radiological outcomes. Rovere et al. highlighted the potential role of blockchain-based systems in orthopedic research by enabling chronologically traceable, secure, and potentially interoperable clinical, imaging, and research data [59]. Such technologies remain developmental and face important technical, regulatory, and implementation challenges, but improved digital data integration may ultimately facilitate higher-quality comparative studies and more reliable future evidence synthesis.

## 5. Limitations

Several limitations should be acknowledged. First, the evidence base included only a small number of RCTs, while retrospective studies contributed substantially to several outcomes. Important directional or magnitude discrepancies between randomized and observational evidence were observed for short-term pain, operative time, cement volume, and kyphosis correction. Although these were addressed through design-stratified analyses, residual confounding and treatment-selection bias remain inherent limitations of the observational evidence. Relevant factors, including age, BMD and osteoporosis severity, fracture level and morphology, baseline vertebral collapse and kyphosis, and clinical status, were inconsistently reported and rarely adjusted for. In particular, surgeon selection of unilateral or bilateral access according to fracture anatomy or technical complexity may have contributed to the observed design-related heterogeneity. Accordingly, RCT findings were given primary inferential weight, while observational evidence and cross-design pooled estimates were interpreted as complementary or exploratory where discordance was present.

Second, fracture acuity and morphology were not uniformly characterized. Although several studies used MRI findings consistent with acute fracture, others did not explicitly report acuity-specific imaging criteria or relied predominantly on symptom duration and clinical history, with variable temporal eligibility windows. Similarly, fracture level, vertebral collapse, posterior wall involvement, intravertebral clefts, baseline kyphosis, and vertebral height loss were incompletely or inconsistently reported. These limitations introduce potential misclassification and residual clinical heterogeneity that could not be explored through subgroup or meta-regression analyses.

Third, procedural and perioperative protocols were not fully standardized across studies. Potential differences in cement viscosity and injection technique, balloon systems, imaging guidance, surgeon experience, anesthesia, postoperative management, and concomitant osteoporosis treatment were incompletely reported and could have influenced procedural, radiographic, and clinical outcomes. In particular, post-fracture anti-osteoporotic management was not consistently reported across studies, precluding assessment of potential differences in bisphosphonate, denosumab, teriparatide, or calcium/vitamin D use between treatment groups; this represents a potential confounder when interpreting subsequent or adjacent vertebral fracture rates.

Fourth, follow-up duration and the definitions of short- and long-term assessment varied across studies, limiting direct temporal comparability. Long-term data were relatively scarce, and several secondary outcomes, including blood loss, hospital stay, loss of kyphotic correction, and specific procedure-related complications, were reported by few studies or using incompatible definitions and metrics, precluding robust quantitative synthesis.

Finally, several analytical limitations should be considered. SDs were imputed for some outcomes when not directly reported, introducing additional uncertainty. Exclusion of influential outliers was based on post hoc sensitivity analyses and should therefore be interpreted cautiously, although these exclusions and their effects were transparently reported. The limited number of studies available for most outcomes also precluded reliable assessment of publication bias. Moreover, the analysis relied on aggregate study-level data rather than individual patient data, preventing adjustment for patient-level differences in fracture morphology, osteoporosis severity, treatment selection, and other potentially relevant effect modifiers.

## 6. Conclusions

The highest-quality randomized evidence indicates that unilateral and bilateral PKP provide broadly comparable clinical efficacy and safety for the treatment of fresh single-level OVCFs, with **no clinically meaningful superiority of either approach demonstrated by the current evidence**. Although the bilateral approach showed statistically significant advantages in selected radiological parameters, including vertebral height restoration and kyphosis correction, these differences were small and of uncertain clinical relevance. Likewise, the unilateral approach was associated with a modest reduction in bone cement volume, without a clearly demonstrated clinically relevant perioperative benefit.

Importantly, **no statistically significant differences were identified in long-term pain relief, functional outcomes, cement leakage rates, or the incidence of adjacent vertebral fractures**. Observational studies provide complementary evidence but show directional discrepancies from randomized trials for several outcomes and should therefore be interpreted cautiously. Overall, the choice between unilateral and bilateral PKP may be individualized according to **vertebral morphology, pedicle anatomy, fracture characteristics, surgeon experience, and technical feasibility**, rather than expectations of superior clinical outcomes. Further large, high-quality randomized controlled trials with standardized outcome reporting and long-term follow-up are warranted to clarify the remaining uncertainties regarding radiological correction and procedure-related complications.

## Figures and Tables

**Table 1:** Cochrane Risk of Bias 2 for the three RCTs.

Study	Randomization Process	Deviations from Intended Interventions	Missing Outcome Data	Measurement of the Outcome	Selection of the Reported Result	Overall Bias
Chung 2008 [43]	● Some concerns	● Some concerns	● Low risk	● Some concerns	● Some concerns	● Some concerns
Tang 2019 [44]	● Some concerns	● Some concerns	● Low risk	● Some concerns	● Some concerns	● Some concerns
Wang 2019 [9] (BMR)	● Low risk	● Low risk	● Some concerns	● Low risk	● Low risk	● Some concerns

Traffic-light legend: ● Low risk ● Some concerns ● High risk.

**Table 2:** ROBINS-I Study-Level Risk of Bias for Retrospective and Prospective Studies

● Serious Risk of Bias			● Moderate Risk of Bias			● Low Risk of Bias		
Study	Confounding	Selection of Participants	Classification of Interventions	Deviations from Intended Interventions	Missing Data	Measurement of Outcomes	Reporting of Results	Overall Risk of Bias
Song 2009 [45]	● Serious	● Moderate	● Low	● Moderate	● Low	● Moderate	● Moderate	● Serious
Liu 2025 [46]	● Moderate	● Moderate	● Low	● Low	● Moderate	● Moderate	● Moderate	● Moderate
Qiao 2023 [15]	● Moderate	● Moderate	● Low	● Low	● Low	● Moderate	● Moderate	● Moderate
Zhang 2021 [47]	● Moderate	● Moderate	● Low	● Low	● Low	● Moderate	● Moderate	● Moderate
Wang 2012 [48]	● Moderate	● Moderate	● Low	● Low	● Low	● Moderate	● Moderate	● Moderate
Qian 2023 [16]	● Moderate	● Moderate	● Low	● Low	● Low	● Moderate	● Moderate	● Moderate
Wan 2020 [49]	● Moderate	● Moderate	● Low	● Low	● Low	● Moderate	● Moderate	● Moderate
Zhang 2022 [50]	● Moderate	● Moderate	● Low	● Low	● Low	● Low	● Low	● Moderate

**Table 3:** Common characteristics of the included studies.

Study	Design	Uni- Pedicular (*n*)	Bi- Pedicular (*n*)	Mean Age	Female (%)	BMD (T-Score)	Vertebral Fracture Levels	Follow-Up
Chung et al., 2008 [43]	RCT	24	28	67.9	94.2	−3.55 (U) −3.64 (B)	T11–L2	Mean 17.2 months (range 12–27)
Song et al., 2009 [45]	Retrospective	15	30	67.6	64.4	−2.83 (U) −2.93 (B)	T6–L4	Approximately 3 months
Wang et al., 2012 [48]	Retrospective	31	31	68.8	51.6	−2.7 (U) −2.9 (B)	T9–L5	16.7 ± 2.5 months (U) 15.9 ± 3.2 months (B)
Tang et al., 2019 [44]	RCT	83	95	73.2	67.4	NR	T11–L2	Mean 9.3 ± 1.8 months (U) 8.5 ± 1.6 months (B)
Wan et al., 2020 [49]	Retrospective	70	68	70.0	63.8	NR	T7–L3	6 months
Wang et al., 2019 [9]	RCT	151	140	68.9	80.4	−3.1 (U) −3.2 (B)	T11–L2	6 months
Zhang et al., 2021 [47]	Retrospective	62	36	76.7	67.3	NR	T6–T10; T11–L2; L3–L5	3 days
Zhang et al., 2022 [50]	Prospective non-randomized	29	38	73.9	62.5	−3.1 (U) −3.2 (B)	Thoracic, thoracolumbar, and lumbar	Mean 17.1 months (range 14–27)
Qian et al., 2023 [16]	Retrospective	82	78	75.1	85.0	NR	L3–L5	2 years
Qiao et al., 2023 [15]	Retrospective	79	68	71.9	78.9	NR	T10–L4	Mean 6.8 months (range 6–8)
Liu et al., 2025 [46]	Retrospective	79	57	72.2	52.9	NR	T11–L2	1 year

**Table 4:** Summary of findings and certainty of evidence.

Outcome	Study Design (No. of Studies)	Effect Estimate (95% CI)	Certainty of Evidence	Key Reasons for Downgrading
Pain (VAS)—Short-term	3 RCTs, 7 retrospectives, 1 prospective (n = 11)	MD −0.02 (−0.18, 0.14); *p* = 0.80	Low	Risk of bias (observational); serious inconsistency (I^2^ = 75.5%, subgroup differences)
Pain (VAS)—Long-term	3 RCTs, 5 retrospectives, 1 prospective (n = 9)	MD 0.01 (−0.11, 0.14); *p* = 0.82	Moderate	Risk of bias (observational); moderate inconsistency (I^2^ = 56.6%)
ODI—Short-term (final model)	1 RCT, 4 retrospectives (n = 5)	MD 0.35 (−0.07, 0.78); *p* = 0.10	Low	Risk of bias; serious imprecision (few studies)
ODI—Long-term (final model)	1 RCT, 3 retrospectives (n = 4)	MD 0.52 (0.09, 0.95); *p* = 0.018	Low	Risk of bias; moderate inconsistency (I^2^ = 33.9%); imprecision
Cement leakage	2 RCTs, 5 retrospectives (n = 7)	OR 1.01 (0.71–1.45); *p* = 0.94	Moderate	Risk of bias (observational) only
New adjacent vertebral fractures	2 RCTs, 5 retrospectives (n = 7)	OR 0.97 (0.61–1.55); *p* = 0.90	Moderate	Risk of bias (observational) only
Operative time	2 RCTs, 6 retrospectives, 1 prospective (n = 9)	MD −10.2 (−16.6, −3.8); *p* = 0.002	Very low	Risk of bias; very serious inconsistency (I^2^ = 98.5%)
Cement volume	2 RCTs, 5 retrospectives, 1 prospective (n = 8)	MD −1.31 (−2.18, −0.44); *p* = 0.003	Very low	Risk of bias; very serious inconsistency (I^2^ = 96.0%)
Vertebral height restoration—Short-term	1 RCT, 2 retrospectives, 1 prospective (n = 4)	MD −0.51 (−0.88, −0.15); *p* = 0.006	Low	Risk of bias; serious imprecision
Vertebral height restoration—Long-term	1 RCT, 1 retrospective, 1 prospective (n = 3)	MD −0.44 (−0.88, −0.005); *p* = 0.048	Very low	Risk of bias; very serious imprecision
Kyphosis correction—Short-term	2 RCTs, 2 retrospectives (n = 4)	MD 0.20 (−0.46, 0.86); *p* = 0.55	Very low	Risk of bias; very serious inconsistency (I^2^ = 74.5%, subgroup differences); imprecision
Kyphosis correction—Long-term	2 RCTs, 1 retrospective (n = 3)	MD 0.94 (−0.56, 2.43); *p* = 0.22	Very low	Risk of bias; very serious inconsistency (I^2^ = 96.0%); very serious imprecision

## Data Availability

The protocol for this meta-analysis has been registered in the PROSPERO database (Registration No: CRD420261341620). The data supporting this meta-analysis have been deposited in the Mendeley Data repository and can be accessed at DOI: 10.17632/rj9d7p8v8k.1.

## Supplementary Materials

The following supporting information can be downloaded at: https://www.mdpi.com/article/10.3390/jcm15187030/s1, Table S1: Fracture_Acuity; Table S2: Fracture_Morphology; Table S3: Newcastle_Ottawa Scale assessment of the included non-randomized studies; Table S4: Individual_Complications; Table S5: VAS_SD_Imputation_Sensitivity; Table S6: Wang2019_ODI_Influence_Diagnostics.

## Abbreviations

The following abbreviations are used in this manuscript:

PKP	Percutaneous kyphoplasty
OVCFs	Osteoporotic vertebral compression fractures
PVP	Percutaneous vertebroplasty
PMMA	Polymethylmethacrylate
VAS	Visual Analog Scale
ODI	Oswestry Disability Index
PRISMA	Preferred Reporting Items for Systematic Reviews and Meta-Analyses
NOS	Newcastle–Ottawa Scale
OR	Odds ratio
MD	Mean difference
REML	Restricted maximum-likelihood
