# Supplementary material for: Unilateral Versus Bilateral Percutaneous Kyphoplasty for Single-Level Thoracolumbar Osteoporotic Vertebral Compression Fractures: A Systematic Review and Meta-Analysis
Source: J Clin Med. 2026 Sep 10;15(18):7030. doi: 10.3390/jcm15187030 (PMC13607866; doi:10.3390/jcm15187030)
Supplement: Supplementary file 1 [file jcm-15-07030-s001.zip › Supplementary_Table_S6_Wang2019_ODI_Influence_Diagnostics.pdf]

Supplementary Table S6. Influence diagnostics for Wang 2019 in ODI meta-analyses

Outcome	Studentised residual	Cook's distance	Change in MD after omission	I^2 reduction after omission	Influential?
ODI short-term	> 2.0	> 1.0	> 0.5 points	> 40 percentage points	Yes
ODI long-term	> 2.0	> 1.0	> 0.5 points	> 40 percentage points	Yes

Abbreviations: ODI, Oswestry Disability Index; MD, mean difference; I^2, heterogeneity statistic.
