# Supplementary material for: Unilateral Versus Bilateral Percutaneous Kyphoplasty for Single-Level Thoracolumbar Osteoporotic Vertebral Compression Fractures: A Systematic Review and Meta-Analysis
Source: J Clin Med. 2026 Sep 10;15(18):7030. doi: 10.3390/jcm15187030 (PMC13607866; doi:10.3390/jcm15187030)
Supplement: Supplementary file 1 [file jcm-15-07030-s001.zip › Supplementary Table S3 Newcastle_Ottawa Scale assessment of the included non randomized studies.pdf]

Supplementary Table S3: Newcastle–Ottawa Scale assessment of the included non-randomized studies

**Study**	**Selection (4)**	**Comparability (2)**	**Outcome (3)**	**Total (9)**
Song 2009	4	0	3	**7/9**
Liu 2025	4	1	2	**7/9**
Qiao 2023	4	1	3	**8/9**
Zhang 2021	4	1	2	**7/9**
Wang 2012	4	1	3	**8/9**
Qian 2023	4	1	3	**8/9**
Wan 2020	4	1	3	**8/9**
Zhang 2022	4	0	3	**7/9**
