# Supplementary material for: Unilateral Versus Bilateral Percutaneous Kyphoplasty for Single-Level Thoracolumbar Osteoporotic Vertebral Compression Fractures: A Systematic Review and Meta-Analysis
Source: J Clin Med. 2026 Sep 10;15(18):7030. doi: 10.3390/jcm15187030 (PMC13607866; doi:10.3390/jcm15187030)
Supplement: Supplementary file 1 [file jcm-15-07030-s001.zip › Supplementary_Table_S1_Fracture_Acuity.pdf]

Supplementary Table S1. Definition and ascertainment of fracture acuity across included studies

Study	Temporal criterion / symptom duration	MRI used	MRI / imaging evidence of acuity	Other diagnostic criteria relevant to fracture classification	Overall ascertainment of fracture acuity
Chung et al., 2008	BK performed within 2 months after injury; mean time from injury 3.44 weeks (U) and 3.68 weeks (B)	NR	NR	Confirmed osteoporotic VCFs at T11-L2; osteoporosis supported by BMD	Primarily temporal/clinical; imaging confirmation of acuity not reported
Song et al., 2009	Pain duration <=6 weeks	NR	NR	Single-level osteoporotic compression fracture; previous or multiple compression fractures excluded; BMD assessed	Primarily temporal/clinical; imaging confirmation of acuity not reported
Wang et al., 2012	<3 months history of low back pain; actual mean duration approximately 23 days (U) and 21 days (B)	NR	NR	Single osteoporotic vertebral fracture; non-osteoporotic fractures excluded; osteoporosis T-values reported	Primarily temporal/clinical; imaging confirmation of acuity not reported
Wang et al., 2019	Poor tolerance of analgesic therapy/bed rest for >=2 weeks; no maximum fracture-duration threshold reported	Yes	Single-level T1-weighted hypointensity, T2-weighted hyperintensity, and STIR hyperintensity	BMD T-score <=-2.5; T11-L2; CT-confirmed AO type A1 fracture	Imaging-confirmed acute osteoporotic fracture
Tang et al., 2019	Actual injury time 1-12 days (U) and 1-15 days (B)	Yes	High signal on fat-suppressed MRI, explicitly described as indicating a fresh fracture; MRI-defined old fractures excluded	Single T11-L2 fracture; X-ray compression 25-75%; CT assessment; BMD T-score <-2.5	Imaging-confirmed fresh osteoporotic fracture
Wan et al., 2020	NR	Yes	MRI required, but no specific marrow-edema or T1/T2/STIR acuity criterion reported	X-ray, CT and MRI used to confirm fracture site and diagnosis; single-segment osteoporotic burst fracture; QCT <80 mg/cm3; AO Spine A3/A4	MRI-confirmed fracture, but fracture acuity not explicitly defined
Zhang et al., 2021	Disease duration <2 weeks; >2 weeks excluded	Yes	MRI/ECT-defined fresh fracture; T1WI low signal, T2WI high/equal signal and STIR high signal; representative cases demonstrated bone marrow edema	BMD-confirmed osteoporosis; single vertebral fracture; no adjacent or distant fractures	Strong temporal and imaging confirmation of fresh osteoporotic fracture
Zhang et al., 2022	Severe OVCF-related pain unresponsive to analgesics for >=2 weeks; no maximum fracture-duration threshold reported	Yes	MRI required for diagnosis of single-level OVCF, but specific MRI signs of acuity not reported	BMD T-score <-2.5; >=15% vertebral height loss; pre- and postoperative 3D CT; pathological fractures excluded	MRI-confirmed OVCF, but acuity not explicitly defined
Qian et al., 2023	PKP performed within 4 weeks of OVCF occurrence; actual mean injury time 4.53 +/- 3.52 days (U) and 4.20 +/- 3.89 days (B)	Yes	T2-weighted STIR showed obvious bone edema in the fractured vertebral body	Single-level OVCF; non-osteoporotic fractures and previous/multiple compression fractures excluded	Strong temporal and MRI confirmation of acute osteoporotic fracture
Qiao et al., 2023	Vertebral compression fracture of <2 weeks duration	Yes	Bone marrow edema with low signal on T1-weighted and high signal on T2-weighted images	BMD T-score <-2.5; single-segment OVCF; secondary osteoporosis and spinal malignancy excluded	Strong temporal and MRI confirmation of acute osteoporotic fracture
Liu et al., 2025	NR; no explicit maximum symptom/fracture duration reported	Yes*	Representative cases demonstrated T2 high signal and T1 low signal at the fracture site and adjacent bone, described as hemorrhagic changes	CT demonstrated a fracture line beneath the superior endplate; BMD T-score <=-2.5; single-level T11-L2 OVCF	Imaging findings compatible with a recent fracture, but acuity was not a formally stated inclusion criterion

Abbreviations: B = bilateral; BMD = bone mineral density; BK = balloon kyphoplasty; CT = computed tomography; ECT = emission computed tomography; MRI = magnetic resonance imaging; NR = not reported; OVCF = osteoporotic vertebral compression fracture; PKP = percutaneous kyphoplasty; QCT = quantitative computed tomography; STIR = short tau inversion recovery; U = unilateral; VCF = vertebral compression fracture.

**In Liu et al. 2025, MRI findings compatible with recent fracture were demonstrated in representative cases, but MRI evidence of acuity was not explicitly stated as a mandatory inclusion criterion.*
