# Supplementary material for: Unilateral Versus Bilateral Percutaneous Kyphoplasty for Single-Level Thoracolumbar Osteoporotic Vertebral Compression Fractures: A Systematic Review and Meta-Analysis
Source: J Clin Med. 2026 Sep 10;15(18):7030. doi: 10.3390/jcm15187030 (PMC13607866; doi:10.3390/jcm15187030)
Supplement: Supplementary file 1 [file jcm-15-07030-s001.zip › Supplementary_Table_S2_Fracture_Morphology.pdf]

Supplementary Table S2. Fracture morphology and baseline radiographic characteristics across included studies

Study	Fracture level / location	Collapse severity / compression	Vertebral height loss / baseline height	Posterior wall involvement	Intravertebral cleft	Baseline kyphotic deformity	Fracture morphology / classification	Relevant information / approach selection
Chung et al., 2008	T11-L2; exact distribution by individual level NR	NR	NR	NR	NR	Cobb angle: 17.6 ± 2.8° (13-25°) U vs 18.5 ± 3.5° (12-27°) B; p=0.369	Osteoporotic VCF; no formal morphological classification reported	Confirmed osteoporosis; mean T-score -3.55 U / -3.64 B. Authors stated that the indication for unilateral and bilateral BK was the same; no morphology-based approach-selection criterion reported.
Song et al., 2009	T6-L4; exact level distribution NR	Preoperative compression ratio: 36.67 ± 13.53% U vs 41.53 ± 14.26% B; p=0.279	Assessed by compression ratio, CR=(A-B)/A×100; separate absolute height-loss value NR	NR	NR	Kyphotic angle: 11.96 ± 5.64° U vs 8.60 ± 4.03° B; p=0.026	Single-level osteoporotic VCF; no formal morphological classification reported	Multiple/previous compression fractures excluded. Bilateral PKP was initially planned in all patients; unilateral treatment resulted when bilateral completion was prevented by intraoperative clinical circumstances rather than fracture morphology.
Wang et al., 2012	U: T10 n=2, T11 n=2, L1 n=6, L2 n=2, L3 n=7, L4 n=3, L5 n=2. B: T9 n=2, T10 n=5, T11 n=6, T12 n=2, L1 n=4, L2 n=6, L3 n=5, L4 n=1*	Baseline collapse quantified using anterior and middle vertebral height relative to the mean height of adjacent vertebrae	Anterior height: 35.6% U vs 31.2% B; middle height: 43.9% U vs 40.1% B; no significant baseline difference reported	NR	NR	NR	Single osteoporotic VCF; no formal AO/Genant or other morphology classification reported	Non-osteoporotic fractures excluded; fractures described as occurring without nerve injury. No morphology-based criterion for unilateral versus bilateral BKP reported.
Wang et al., 2019	T11-L2 single-level fractures	Inclusion required 15-70% anterior vertebral compression	Baseline anterior vertebral height ratio: 48.2 ± 12.8% U vs 46.6 ± 13.0% B; p=0.66	CT-confirmed AO type A1 morphology; posterior-wall involvement was not separately reported as a binary variable	NR	Preoperative kyphotic angle: 15.3 ± 5.7° U vs 15.8 ± 5.2° B; p=0.74	CT-confirmed AO type A1 fracture; MRI additionally required single-level T1↓, T2↑ and STIR↑ signal	BMD T-score ≤-2.5. Fracture morphology was relatively restricted by eligibility criteria. No reported morphology-based criterion for assigning unilateral versus bilateral PKP.
Tang et al., 2019	T11-L2. U: T11 n=9, T12 n=32, L1 n=27, L2 n=15; B: T11 n=7, T12 n=37, L1 n=33, L2 n=18	Inclusion required 25-75% vertebral compression on X-ray	Preoperative anterior height: 16.4 ± 1.8 mm U vs 15.9 ± 1.7 mm B; p=0.058. Middle height: 15.3 ± 1.4 mm U vs 15.7 ± 1.6 mm B; p=0.080	Intact posterior wall required on preoperative CT; incomplete burst fractures/posterior-wall compression with neurological compression excluded	NR	Preoperative Cobb angle: 34.3 ± 3.5° U vs 33.8 ± 3.4° B; p=0.336	Single-level thoracolumbar OVCF; no formal AO/Genant classification reported; fresh fracture confirmed by fat-suppressed MRI	Authors discussed bilateral puncture as preferable for severe compression and unilateral puncture when collapse is predominantly unilateral; these were recommendations, not reported allocation criteria for the randomized cohort.
Wan et al., 2020	T7-L3. U: T7 2, T8 0, T9 4, T10 0, T11 10, T12 24, L1 18, L2 10, L3 2; B: T7 0, T8 2, T9 4, T10 4, T11 12, T12 18, L1 18, L2 10, L3 2	Inclusion required anterior vertebral body height collapse ≤2/3	Mean preoperative vertebral height: 17.05 ± 1.94 mm U vs 16.71 ± 2.18 mm B; no significant baseline difference	Yes - burst-fracture morphology with posterior vertebral wall disruption; intraspinal occupancy <25% required; no neurological deficit	NR	Preoperative Cobb angle: 19.25 ± 3.46° U vs 20.26 ± 3.21° B; no significant baseline difference	AO Spine A3/A4 osteoporotic thoracolumbar burst fractures. U: A3 n=40, A4 n=30; B: A3 n=36, A4 n=32	Severe PLC injury/fracture-dislocation excluded. In unilateral PKP, puncture side and angle were individualized according to vertebral morphology, with the balloon positioned slightly toward the compression side.
Zhang et al., 2021	T6-T10: n=20; T11-L2: n=62; L3-L5: n=16; group-specific counts NR	NR; representative cases described wedge-shaped changes, but no quantitative compression threshold or formal collapse grade reported	Preoperative anterior vertebral body height: 19.6 ± 6.5 mm U vs 19.2 ± 6.1 mm B; no significant intergroup difference reported	Incomplete posterior wall with spinal cord/nerve-root compression symptoms excluded; intact posterior wall was not required for all included fractures	NR	NR	Single-level fresh osteoporotic VCF; representative cases show wedge-shaped fractures, but no formal AO/Genant classification was applied	Adjacent/distant fractures excluded. Authors noted that fracture type should be considered when choosing unilateral versus bilateral puncture, but actual morphology-based allocation criteria were not reported.
Zhang et al., 2022	Single-level OVCF required; treated vertebral-level distribution NR	Inclusion required ≥15% height loss of the fractured vertebra	Preoperative mean VH: 16.2 ± 2.4 mm U vs 16.2 ± 1.9 mm B; VH measured on 3D CT as mean anterior, middle and posterior vertebral height	Not specifically reported; spinal cord/nerve-root compression excluded, which does not establish absence of posterior-wall involvement	NR	NR	Single-level OVCF diagnosed by MRI; no formal AO/Genant or other morphological classification reported	All patients underwent preoperative 3D CT. Prospective nonrandomized study; no explicit morphology-based criteria for assignment to unilateral versus bilateral PKP reported.
Qian et al., 2023	Lower lumbar L3-L5. U: L3 n=34, L4 n=31, L5 n=17; B: L3 n=32, L4 n=32, L5 n=14; p=0.875	Authors described the degree of compression as relatively mild/unremarkable; no explicit percentage-of-collapse eligibility threshold	AVBHr: 85.46 ± 10.26% U vs 85.69 ± 11.22% B (p=0.892); MVBHr: 80.32 ± 13.81% vs 77.26 ± 12.81% (p=0.150); PVBHr: 90.87 ± 8.91% vs 88.81 ± 9.96% (p=0.105)	NR; neurological deficits excluded, but this does not establish absence of posterior-wall involvement	NR; discussed only as a general risk factor for cement leakage, not reported for the cohort	NR	EVOSG: A (wedging) 25 U / 26 B; B (biconcavity) 47 U / 44 B; C (crush) 10 U / 8 B; p=0.888	Single-level acute OVCF; severe spinal deformity and neurological deficits excluded. No significant baseline differences in segmental distribution or fracture morphology.
Qiao et al., 2023	T10-L4; overall: T10 n=14, T11 n=19, T12 n=25, L1 n=31, L2 n=28, L3 n=19, L4 n=11; no significant U/B difference	Compression fracture required; no quantitative percentage/grade of collapse reported. In unilateral PKP, puncture side was selected preferentially on the side with more severe compression or more pronounced pain	Preoperative anterior vertebral body height: 16.45 ± 2.34 mm U vs 16.87 ± 1.60 mm B; p=0.571	NR; fractures producing neurological symptoms excluded, which does not establish absence of posterior-wall involvement	NR	Preoperative Cobb angle: 23.73 ± 2.57° U vs 24.11 ± 1.81° B; p=0.649	Single-segment acute OVCF with MRI-confirmed bone marrow edema; no formal morphological classification reported	BMD T-score <-2.5. Within the unilateral group, puncture side was individualized according to asymmetry of vertebral compression and/or pain.

Study	Fracture level / location	Collapse severity / compression	Vertebral height loss / baseline height	Posterior wall involvement	Intravertebral cleft	Baseline kyphotic deformity	Fracture morphology / classification	Relevant information / approach selection
Liu et al., 2025	T11-L2. U: T11 n=17, T12 n=23, L1 n=28, L2 n=11; B: T11 n=8, T12 n=24, L1 n=14, L2 n=11; p=0.21	OVCF with vertebral compression required; no quantitative collapse-severity threshold reported	Preoperative vertebral height: 18.49 ± 4.63 mm U vs 18.81 ± 3.93 mm B; p=0.67	Intact posterior vertebral wall required, with no retropulsed bony fragments into the spinal canal	NR	Preoperative local kyphotic angle: 23.05 ± 7.21° U vs 23.20 ± 6.08° B; p=0.89	Single-level T11-L2 osteoporotic compression fracture; no formal AO/Genant/EVOSG classification reported	Preexisting spinal deformity excluded. Unilateral access targeted the more affected/more collapsed side. Surgeon preference/intraoperative judgment influenced approach; narrow pedicles or asymmetric fractures could favor bilateral access, and inadequate unilateral filling could prompt second-pedicle cannulation.

Abbreviations: AVBHr = anterior vertebral body height ratio; B = bilateral; BMD = bone mineral density; BK/BKP = balloon kyphoplasty; CT = computed tomography; EVOSG = European Vertebral Osteoporosis Study Group; MVBHr = middle vertebral body height ratio; NR = not reported; OVCF = osteoporotic vertebral compression fracture; PKP = percutaneous kyphoplasty; PLC = posterior ligamentous complex; PVBHr = posterior vertebral body height ratio; STIR = short tau inversion recovery; U = unilateral; VCF = vertebral compression fracture; VH = vertebral height.

**The individual vertebral-level counts reported for the unilateral group in Wang et al. (2012) sum to 24 rather than the stated group size of 31; values are reproduced as reported in the primary article and were not imputed or corrected.*
