# Supplementary material for: Unilateral Versus Bilateral Percutaneous Kyphoplasty for Single-Level Thoracolumbar Osteoporotic Vertebral Compression Fractures: A Systematic Review and Meta-Analysis
Source: J Clin Med. 2026 Sep 10;15(18):7030. doi: 10.3390/jcm15187030 (PMC13607866; doi:10.3390/jcm15187030)
Supplement: Supplementary file 1 [file jcm-15-07030-s001.zip › Supplementary_Table_S4_Individual_Complications.pdf]

Supplementary Table S4. Individual procedure-related complications by study and treatment arm

Study	Unilateral PKP, n	Bilateral PKP, n	Individual complications – Unilateral PKP	Individual complications – Bilateral PKP	Relevant details
Chung et al., 2008	24	28	PMMA leakage into spinal canal: 2/24; neurological deficit: 0/24	No complications reported	Both spinal-canal leakages were asymptomatic. Possible medial pedicle-wall violation was suggested as a technical mechanism.
Song et al., 2009	15	30	Nerve injury: 0/15; pedicle fracture: 0/15	Nerve injury: 0/30; pedicle fracture: 0/30	No postoperative procedural complications were reported. Intraoperative events preventing completion of initially planned bilateral PKP were not considered complications attributable to unilateral PKP.
Wang et al., 2012	31	31	Cement leakage: 4/31; pedicle fracture: 0/31; nerve damage: 0/31; pulmonary embolism: 0/31	Cement leakage: 5/31; pedicle fracture: 0/31; nerve damage: 0/31; pulmonary embolism: 0/31	Cement leakage occurred into paravertebral soft tissues or the intervertebral disc. No pulmonary embolism was observed.
Wang et al., 2019	151	140	Cement leakage: 36/151; pulmonary cement embolism: 1/151; nerve root stimulation: 16/151	Cement leakage: 33/140; pulmonary cement embolism: 1/140; nerve root stimulation: 3/140	Pulmonary cement emboli were asymptomatic. Nerve root stimulation was significantly more frequent with unilateral PKP ($p<0.05$). Greater medial angulation and repeated needle-position adjustment were proposed as possible mechanisms.
Tang et al., 2019	83	95	Cement leakage: 0/83; pulmonary embolism: 0/83; nerve injury: 0/83; infection: 0/83	Cement leakage: 0/95; pulmonary embolism: 0/95; nerve injury: 0/95; infection: 0/95	No obvious intraoperative bone-cement reaction was reported.
Wan et al., 2020	70	68	Cement leakage: 8/70 (11.4%); incision swelling: 19.71%	Cement leakage: 7/68 (10.3%); incision swelling: 30.88%	No statistically significant between-group difference was reported for these events.
Zhang et al., 2021	62	36	NR	NR	Individual procedure-related adverse events were not quantitatively reported by treatment arm.
Zhang et al., 2022	29	38	NR	NR	Individual procedure-related adverse events were not quantitatively reported by treatment arm.
Qian et al., 2023	82	78	Cement leakage: 19/82 (23.2%)	Cement leakage: 20/78 (25.6%)	No statistically significant between-group difference in cement leakage was reported. Other individual complications were not quantitatively reported by treatment arm.
Qiao et al., 2023	79	68	Cement leakage: 7/79 (8.9%); puncture-related postoperative pain: 3/79	Cement leakage: 5/68 (7.4%); puncture-related postoperative pain: 15/68	All cement leakages were asymptomatic. Puncture-related pain was significantly more frequent after bilateral PKP ($p<0.05$) and resolved within 1 week with conservative treatment. No cardiovascular or cerebrovascular intraoperative adverse events occurred.
Liu et al., 2025	79	57	Cement leakage: 4/79 (5.1%); transient intercostal nerve irritation: 1/79; pulmonary cement embolism: 0/79; deep infection: 0/79	Cement leakage: 3/57 (5.3%); paravertebral muscle hematoma: 1/57; pulmonary cement embolism: 0/57; deep infection: 0/57	Cement extravasations were asymptomatic. Nerve irritation resolved spontaneously within 2 weeks; hematoma was treated conservatively. No severe procedure-related complications were reported.

Abbreviations: PKP, percutaneous kyphoplasty; PMMA, polymethylmethacrylate; NR, not reported.

Note: Only complications explicitly reported in the primary studies are presented. NR indicates that individual complication data were not quantitatively reported by treatment arm and should not be interpreted as zero events. Subsequent or adjacent vertebral fractures were excluded because they were analyzed separately as a predefined outcome in the present meta-analysis.
