# Supplementary material for: Unilateral Versus Bilateral Percutaneous Kyphoplasty for Single-Level Thoracolumbar Osteoporotic Vertebral Compression Fractures: A Systematic Review and Meta-Analysis
Source: J Clin Med. 2026 Sep 10;15(18):7030. doi: 10.3390/jcm15187030 (PMC13607866; doi:10.3390/jcm15187030)
Supplement: Supplementary file 1 [file jcm-15-07030-s001.zip › Supplementary_Table_S5_VAS_SD_Imputation_Sensitivity.pdf]

Supplementary Table S5. Sensitivity analyses for VAS outcomes after excluding studies with imputed standard deviations

Outcome	Analysis	k	MD (95% CI)	p	I^2
VAS short-term	All studies	11	−0.02 (−0.18, 0.14)	0.80	75.5%
VAS short-term	Excluding imputed SD studies	9	−0.045 (−0.215, 0.125)	0.60	79.3%
VAS long-term	All studies	9	0.01 (−0.11, 0.14)	0.82	56.6%
VAS long-term	Excluding imputed SD studies	7	0.020 (−0.118, 0.157)	0.78	64.3%

Abbreviations: VAS, Visual Analog Scale; SD, standard deviation; MD, mean difference; CI, confidence interval; k, number of studies; I^2, heterogeneity statistic.
